# *N*^6^-methyladenosine modification-mediated mRNA metabolism is essential for human pancreatic lineage specification and islet organogenesis

**DOI:** 10.1038/s41467-022-31698-2

**Published:** 2022-07-18

**Authors:** Xiaojie Ma, Jie Cao, Ziyu Zhou, Yunkun Lu, Qin Li, Yan Jin, Guo Chen, Weiyun Wang, Wenyan Ge, Xi Chen, Zhensheng Hu, Xiao Shu, Qian Deng, Jiaqi Pu, Chengzhen Liang, Junfen Fu, Jianzhao Liu, Saiyong Zhu

**Affiliations:** 1 MOE Key Laboratory of Biosystems Homeostasis & Protection and Zhejiang Provincial Key Laboratory for Cancer Molecular Cell Biology, Life Sciences Institute, Zhejiang University, Hangzhou, Zhejiang 310058 China; 2 MOE Key Laboratory of Macromolecular Synthesis and Functionalization, Department of Polymer Science and Engineering, Zhejiang University, Hangzhou, 310008 China; 3 Life Sciences Institute, Zhejiang University, Hangzhou, 310058 China; 4 The Children’s Hospital, Zhejiang University School of Medicine, Hangzhou, 310003 China; 5 Department of Orthopedics Surgery, the Second Affiliated Hospital, School of Medicine, Zhejiang University, Hangzhou, 310009 China

**Keywords:** Stem-cell differentiation, RNA, Organogenesis, RNA modification

## Abstract

Pancreatic differentiation from human pluripotent stem cells (hPSCs) provides promising avenues for investigating development and treating diseases. *N*^6^-methyladenosine (m^6^A) is the most prevalent internal messenger RNA (mRNA) modification and plays pivotal roles in regulation of mRNA metabolism, while its functions remain elusive. Here, we profile the dynamic landscapes of m^6^A transcriptome-wide during pancreatic differentiation. Next, we generate knockout hPSC lines of the major m^6^A demethylase *ALKBH5*, and find that ALKBH5 plays significant regulatory roles in pancreatic organogenesis. Mechanistic studies reveal that ALKBH5 deficiency reduces the mRNA stability of key pancreatic transcription factors in an m^6^A and YTHDF2-dependent manner. We further identify that ALKBH5 cofactor α-ketoglutarate can be applied to enhance differentiation. Collectively, our findings identify ALKBH5 as an essential regulator of pancreatic differentiation and highlight that m^6^A modification-mediated mRNA metabolism presents an important layer of regulation during cell-fate specification and holds great potentials for translational applications.

## Abstract (web summary)

Ma et al. profile the dynamic landscape of m^6^A during pancreatic differentiation, and identify ALKBH5 as an essential m^6^A regulator supporting pancreatic differentiation, indicating a role for m^6^A-mediated mRNA metabolism in cell-fate specification.

## Introduction

In the last decades, model organisms, such as zebrafish, frog, and mouse, have been used to describe the normal development of the pancreas^1^. These studies have identified an intricate regulatory network containing transcription factors, epigenetic regulators, and signaling pathways that can orchestrate pancreatic differentiation from hPSCs^2^. It is necessary to understand how human pancreatic lineages are specified and use that information to make functional human pancreatic cells that can be applied to interpret and treat metabolic diseases, including diabetes. However, the technical and ethical challenges remain daunting in the study of early human development. In recent years, tremendous efforts have been made to differentiate hPSCs into insulin-secreting pancreatic β-like cells^3–12^. Briefly, stepwise differentiation protocols have been developed to guide the directed differentiation of hPSCs into definitive endoderm (DE), posterior foregut (PF), pancreatic progenitors (PPs), and finally human islet-like organoids (hILOs). Now, the hPSC-based pancreatic differentiation represents a feasible platform to study human pancreatic biology. In addition, precise genome editing and high-throughput sequencing approaches have provided researchers powerful tools to investigate pancreatic differentiation and development^13–15^. Recently, such approaches have been successfully applied to study the roles of many key pancreatic development and diabetic susceptibility genes, including *PDX1*, *NGN3*, *RFX6*, *GATA6*, *GLIS3*, *HNF1A*, and *MAFB*^16–21^. These studies demonstrate that hPSCs can offer a unique opportunity for investigating human development and disease phenotypes.

*N*^6^-methyladenosine (m^6^A) is the most prevalent internal messenger RNA (mRNA) modification in mammals^22,23^. The mRNA m^6^A modification is installed by a large mRNA methyltransferase complex composed of METTL3, METTL14, WTAP, and auxiliary or scaffold proteins like VIRMA, ZC3H13, and HAKAI^24–26^. The m^6^A mark can be dynamically removed by the demethylases FTO and ALKBH5^27,28^. Multiple m^6^A reader proteins, including YTHDF1-3, YTHDC1-2, and IGF2BP1-3, can specifically bind to m^6^A sites and implement their biological functions^29–33^. The mRNA m^6^A modification is highly dynamic, and influences mRNA metabolism, including their processing, export, stability, and translation. Therefore, mRNA m^6^A modification plays a very important physiological roles. Recent studies suggest that m^6^A modification of mRNAs regulates various biological processes, including circadian rhythms, spermatogenesis, neurogenesis, pluripotency, and immunity^34^. Furthermore, increasing evidence supports the pathological roles of perturbed m^6^A metabolism in several diseases, such as tumorigenesis and inflammation^35,36^. However, the roles of m^6^A modification-mediated mRNA metabolism in pancreatic differentiation and development remain elusive.

ALKBH5 belongs to the AlkB family of α-ketoglutarate-dependent dioxygenases and is a major m^6^A demethylase. Zheng et al. identified ALKBH5 as an m^6^A eraser and demonstrated that *Alkbh5* deficiency led to aberrant spermatogenesis and apoptosis in mouse testes^28^. Subsequently, Zhang et al. found that ALKBH5 was responsible for the hypoxia-induced breast cancer stem cell phenotype^37^. Similarly, Zhang et al. showed that ALKBH5 maintained tumorigenicity of glioblastoma stem-like cells by sustaining *FOXM1* expression and cell proliferation program^38^. Liu et al. identified that ALKBH5 modulated cellular metabolic rewiring and inhibited viral replication^39^. Later, Zhang et al. demonstrated that ALKBH5 promoted the cell proliferation of renal cell carcinoma by regulating *AURKB* expression^40^. Recently, Shen et al. and Wang et al. independently found that ALKBH5 played important roles in acute myeloid leukemia and could be harnessed for effective therapy^41,42^. In addition, Li et al. found that ALKBH5 regulated anti-PD-1 therapy response by modulating tumor microenvironment^43^. Besides tumorigenesis, the functions of ALKBH5 in other biological processes are still largely unknown. Until now, the roles of ALKBH5 in pancreatic differentiation have not been studied.

In this work, we employ hPSC-based pancreatic differentiation platform to profile the mRNA m^6^A dynamics during pancreatic differentiation, and find that m^6^A demethylase ALKBH5 plays essential roles in pancreatic differentiation and islet organogenesis by modulating m^6^A and YTHDF2-dependent mRNA metabolism.

## Results

### mRNA m^6^A landscape during human pancreatic differentiation

**Fig. 1:**
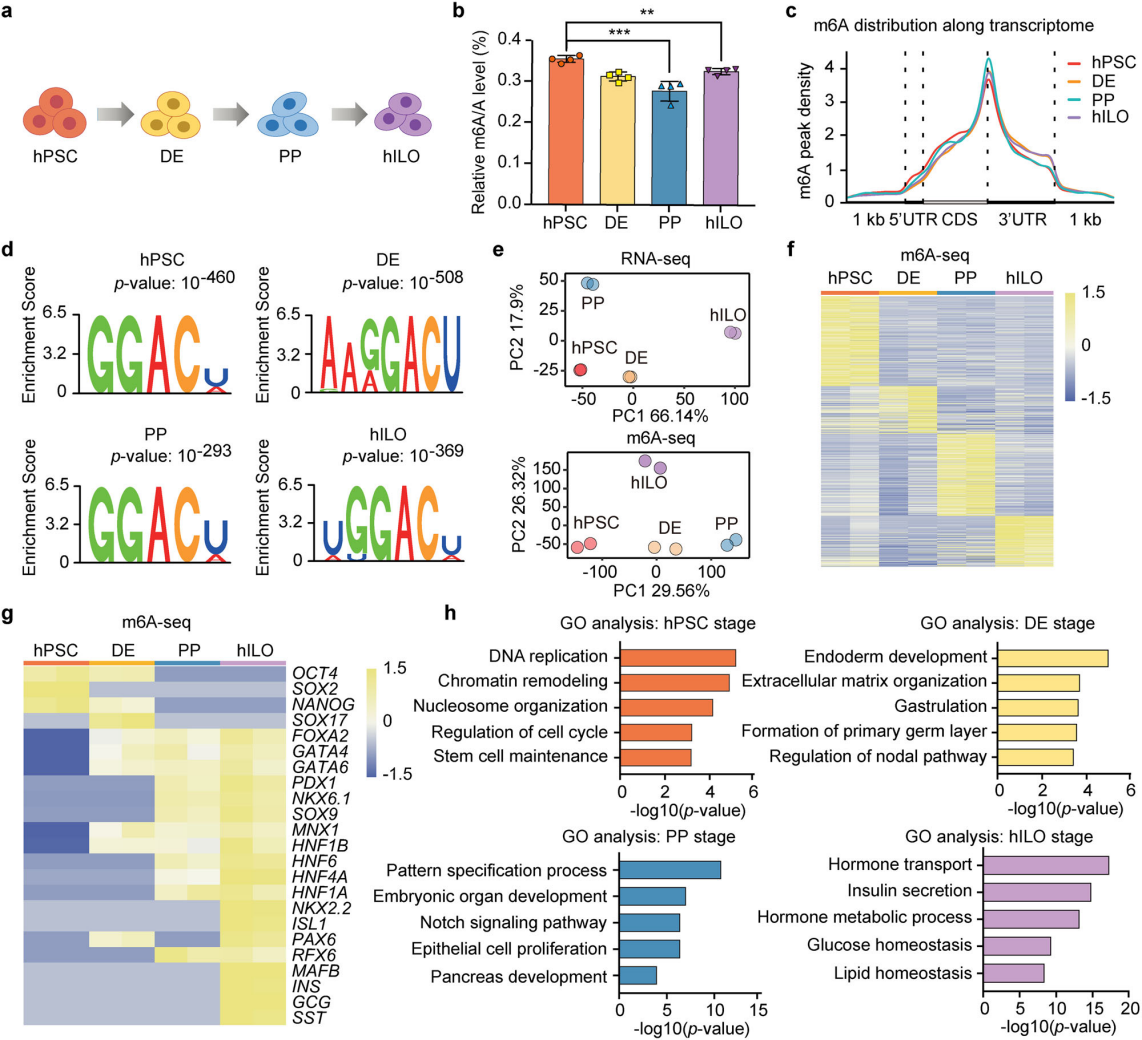
mRNA m^6^A modification is highly dynamic during pancreatic differentiation from hPSCs. **a** A schematic representation of the pancreatic differentiation process from hPSCs to hILOs. **b** The quantification of global m^6^A abundance in hPSCs, DE, PPs, and hILOs by UHPLC-QQQ-MS/MS (*n* = 4 biological replicates). Data are presented as mean ± s.d. Statistical significance calculated using two-tailed Student’s *t*-test, ** *p* < 0.01, *** *p* < 0.001. **c** m^6^A peak distributions along transcripts in hPSCs, DE, PPs, and hILOs. **d** Representative m^6^A motifs in hPSCs, DE, PPs, and hILOs. *p*-values were calculated using one-tailed hypergeometric test. **e** Principal component analysis (PCA) of RNA-seq and m^6^A-seq data. **f** Heatmap visualization of stage-specific upregulated m^6^A peak abundance in hPSCs, DE, PPs, and hILOs. **g** Heatmap visualization for m^6^A abundance of stage-enriched marker genes. **h** Gene ontology (GO) analysis of stage-specific upregulated genes with m^6^A modification, *p*-values were calculated by one-tailed hypergeometric test. Source data are provided as a Source Data file.

To investigate mRNA m^6^A dynamics during human pancreatic differentiation, we step-wisely differentiated hPSCs into human islet-like organoids (hILOs) (Fig. 1a; Supplementary Fig. 1a). We collected samples from hPSC, DE, PP, and hILO stages, and then quantified the mRNA m^6^A levels by high performance liquid chromatography coupled with triple quadrupole tandem mass spectrometry (UHPLC-QQQ-MS/MS). The m^6^A level of mRNAs was significantly downregulated in PPs (0.28% on average) compared to those in hPSCs, DE, and hILOs (0.35%, 0.31%, and 0.33%, respectively) (Fig. 1b). This interesting observation directed us to use the hPSC-based pancreatic differentiation platform to further study the dynamics of mRNA m^6^A modification during cell-fate specification.

To explore the roles of mRNA m^6^A mark in gene expression during pancreatic lineage specification in more details, we performed m^6^A RNA immunoprecipitation (RIP) with next generation sequencing (m^6^A-seq) and RNA-seq on collected samples from the hPSC, DE, PP, and hILO stages. It is a challenging task to profile differentiated cell samples with limited input RNA materials. We applied a modified m^6^A-seq protocol^44^, started with 2−10 μg total RNA, and successfully prepared the sequencing libraries. Correlation analysis between replicates showed similar pattern between m^6^A-seq and RNA-seq data, and the results were reproducible with high correlation between two biological replicates from the same stages (Supplementary Fig. 1b). About 80% transcripts contained m^6^A modification in these four cell populations (Supplementary Fig. 1c). Additionally, the m^6^A level notably decreased at PP stage according to m^6^A-seq (Supplementary Fig. 1d, e), which is consistent with the result of UHPLC-QQQ-MS/MS (Fig. 1b). The majority of m^6^A peaks were distributed in the coding sequence (CDS) and 3’-untranslated regions (3’UTR) (Fig. 1c). The motif enrichment analysis of m^6^A peaks showed that canonical m^6^A motif DRACH (D = A, G, U; R = A, G; H = A, C, U) was dominant in all samples (Fig. 1d). The principal component analysis (PCA) and heatmap results revealed the dynamic changes of m^6^A modifications during pancreatic specification, which were correlated with the gene expression pattern at each stage (Fig. 1e–g; Supplementary Fig. 1f). For example, at hPSC stage, we detected m^6^A peaks specifically at the loci of pluripotent marker genes *OCT4*, *SOX2*, and *NANOG*; at DE stage, we detected m^6^A peaks at the gene loci of *SOX17* and *FOXA2*; at PP stage, m^6^A modifications are emerged at the gene loci of *PDX1*, *NKX6.1*, *SOX9*, *MNX1*, and *HNF6*; at hILO stage, we could observe m^6^A peaks at the gene loci of *INS*, *GCG*, and *SST* (Fig. 1g; Supplementary Fig. 1g). These data imply that m^6^A modification may participate in regulating marker gene expression during pancreatic differentiation. Gene ontology (GO) analysis demonstrated that m^6^A-tagged mRNAs have vital functions in multiple biological processes: DNA replication and stem cell maintenance at hPSC stage; endoderm development as well as gastrulation at DE stage; epithelial cell proliferation and pancreas development at PP stage; insulin secretion and glucose homeostasis at hILO stage (Fig. 1h). These results highlight the dynamics of m^6^A modification in mRNAs during pancreatic specification and suggest that the rewiring of m^6^A modification modulates gene expression upon transition of hPSCs to hILOs.

### mRNA m^6^A dynamics across human pancreatic differentiation

**Fig. 2:**
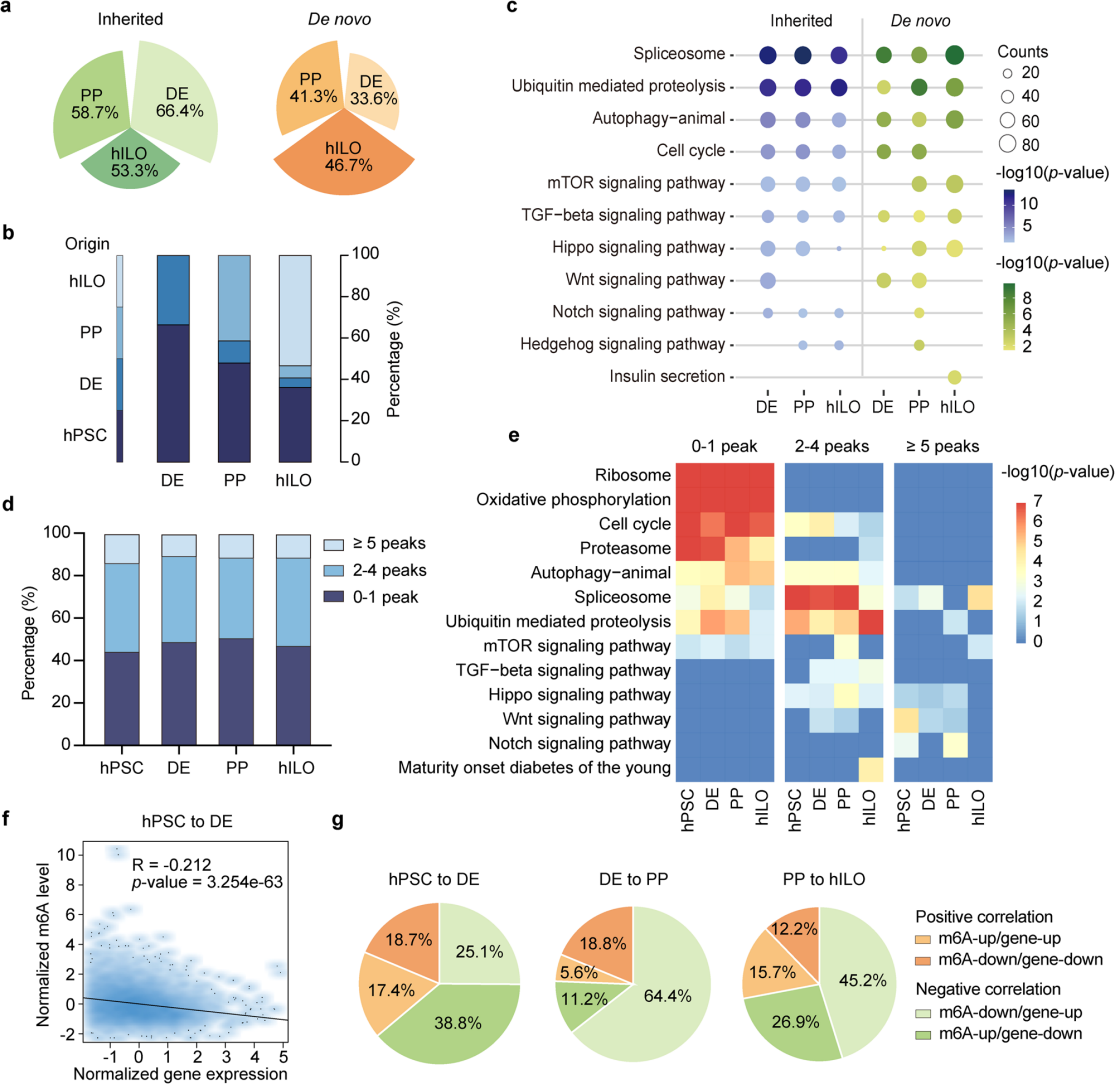
mRNA m^6^A modification regulates function acquiring across pancreatic specification. **a** Nightingale rose diagram showing the percentage of m^6^A peaks inherited from the previous stage or de novo arose at current stage. **b** Stacked histogram showing the origin of m^6^A peaks at stages in pancreatic differentiation. **c** Bubble plots of KEGG pathways enriched in inherited and de novo established m^6^A peaks related genes in DE, PPs, and hILOs. Cycle size represents the gene numbers in each pathway, blue or green gradient represents the *p*-value of each pathway, *p*-values were calculated by one-tailed hypergeometric test. **d** Stacked histogram showing the percentage of genes with different numbers of m^6^A peaks in hPSCs, DE, PPs, and hILOs. **e** Heatmap of KEGG pathways enriched in genes with different numbers of m^6^A peaks in hPSCs, DE, PPs, and hILOs, *p*-values were calculated by one-tailed hypergeometric test. **f** Density scatter plot showing the correlation of gene expression and m^6^A level from hPSCs to DE, *p*-value was calculated by two-tailed hypothesis test. **g** Pie charts showing the specific correlation between mRNA levels and m^6^A abundance from hPSCs to hILOs.

To further investigate the dynamics of m^6^A modifications during pancreatic differentiation, we analyzed the origin of m^6^A modifications, which can inherit from earlier differentiation stages or yield de novo^45^. Intriguingly, the percentage of inherited peaks gradually decreased and the percentage of de novo peaks gradually increased during pancreatic differentiation (Fig. 2a). In PP and hILO stage, most of the inherited peaks originated from hPSCs (Fig. 2b). The Kyoto encyclopedia of genes and genomes (KEGG) analysis showed that genes with inherited m^6^A peaks were more related to fundamental biological functions, such as spliceosome, ubiquitin-mediated proteolysis, autophagy, and cell cycle (Fig. 2c). On the other hand, genes contained de novo m^6^A peaks were more dynamic, and related to signaling pathways and stage-specific functions (Fig. 2c). Next, we clustered mRNAs into three categories according to m^6^A-tagged peak number (0–1 peak, 2–4 peaks, and ≥5 peaks) and observed similar distribution pattern among stages (Fig. 2d). GO and KEGG analyses showed that transcripts with more m^6^A peaks were also more dynamic and enriched in various signaling pathways (e.g., Wnt, TGFβ, and Notch) (Fig. 2e; Supplementary Fig. 2a). These results indicate that mRNA m^6^A modification becomes stage-specific and more diverse, contributing to pancreatic specification.

To evaluate the relationship between m^6^A modification and mRNA expression level, we next performed an integrated and stepwise analysis of the m^6^A-seq and RNA-seq datasets. Globally, the negative correlations between m^6^A abundance and mRNA levels were observed among the transitions from hPSCs to hILOs (Fig. 2f, g; Supplementary Fig. 2b). Of note, we observed the highest percentage of upregulated genes with m^6^A downregulation during the DE-to-PP transition, which is consistent with the significant reduction of m^6^A level at PP stage. These data further suggest that m^6^A modifications play a critical role in regulating mRNA stability and degradation.

**Fig. 3:**
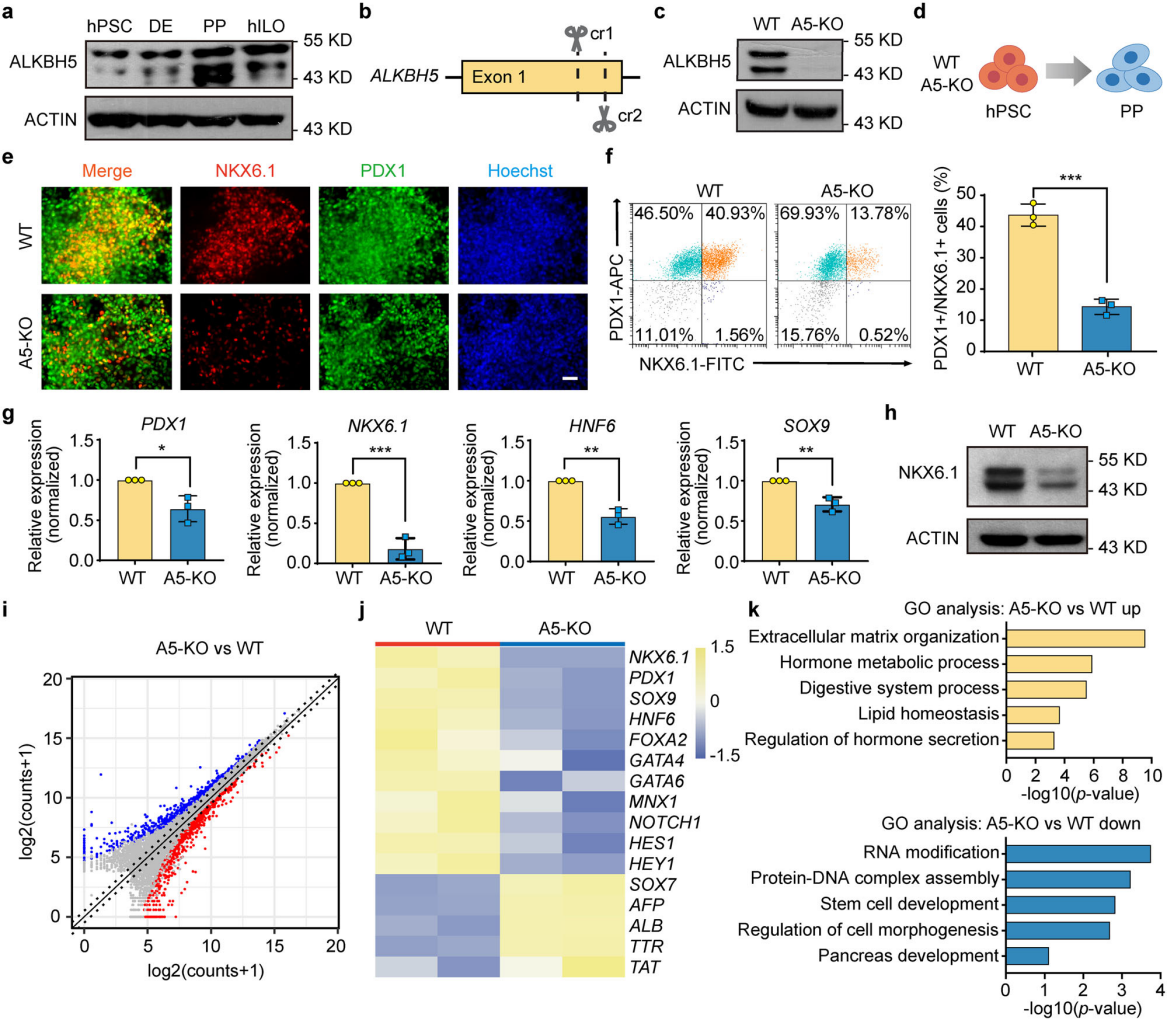
ALKBH5 is essential for pancreatic lineage specification. **a** Western blotting analysis of ALKBH5 protein levels in hPSCs, DE, PPs, and hILOs. ACTIN was used as a loading control. Images are representative of three independent replicates. **b** A schematic representation of the generation of A5-KO hPSCs using CRISPR-Cpf1. **c** Western blotting analysis of ALKBH5 protein levels in WT and A5-KO hPSCs. Images are representative of three independent replicates. **d** A schematic representation of the differentiation of WT and A5-KO hPSCs to PP stage. **e** Immunofluorescent staining of WT and A5-KO PPs for PDX1, NKX6.1, and nuclei (Hoechst). Images are representative of six independent replicates. Scale bar, 100 μm. **f** Representative flow cytometry plots and the percentage of PDX1 and NKX6.1 double-positive PPs in WT and A5-KO populations (*n* = 3 biological replicates). **g** The expressions of PP marker genes *PDX1*, *NKX6.1*, *HNF6*, and *SOX9* in WT and A5-KO PPs (*n* = 3 biological replicates). **h** Representative western blotting of NKX6.1 protein in WT and A5-KO PPs. ACTIN was used as a loading control. Images are representative of three independent replicates. **i** Volcano plot of differentially expressed genes in A5-KO PPs versus WT PPs. Red, upregulated genes (*n* = 965, FC > 1.5, *p* < 0.05); blue, downregulated genes (*n* = 811, FC < 0.67, *p* < 0.05). **j** Heatmap visualization for the expression of upregulated and downregulated marker genes. **k** GO analysis of upregulated and downregulated genes, *p*-values were calculated by one-tailed hypergeometric test. All data are expressed as mean ± s.d. Statistical significance calculated using two-tailed Student’s *t*-test, * *p* < 0.05, ** *p* < 0.01, *** *p* < 0.001. Source data are provided as a Source Data file.

These results led us to investigate how m^6^A modification functionally participate in pancreatic differentiation. Interestingly, among the m^6^A core regulators, the expression of *ALKBH5* was upregulated from PP stage, which was inversely correlated with the m^6^A level change during pancreatic differentiation (Fig. 1b; Supplementary Figs. 1d, e, 2c, d). The protein level of ALKBH5 also increased at PP stage (Fig. 3a). So far, *ALKBH5* knockout (KO) hPSCs and the roles of ALKBH5 in hPSC differentiation have not been reported. Inspired by the above intriguing observations, we therefore decided to explore the roles of ALKBH5 in human pancreatic differentiation.

### Generation of *ALKBH5* knockout hPSC lines

Using our recently established CRISPR-Cpf1 system for precise genome editing of hPSCs^13^. we generated *ALKBH5* KO hPSC lines (Fig. 3b; Supplementary Fig. 3a). We designated *ALKBH5* KO hPSCs as A5-KO, and wild type hPSCs as WT. The PCR genotyping and Sanger sequencing results suggested the success in the generation of *ALKBH5* KO cell lines (A5-KO and A5-KO2) (Supplementary Figs. 3b, c, 4a). Next, western blotting results verified the absence of ALKBH5 proteins in the homozygous A5-KO lines (Fig. 3c; Supplementary Fig. 4b).

A5-KO hPSCs have typical hPSC morphology and proliferate normally during long-term in vitro culture. Immunostaining results showed that both WT and A5-KO hPSCs almost homogenously expressed pluripotent stem cell markers OCT4 and NANOG (Supplementary Figs. 3d, 4c). Using RT-qPCR, we did not detect the significant differences of the marker genes *OCT4*, *SOX2*, and *NANOG* expression levels between WT and A5-KO hPSCs (Supplementary Fig. 3e). These results suggest that ALKBH5 is not required for hPSC survival and self-renewal.

### ALKBH5 is required for pancreatic lineage specification

We checked the differentiation capacity of WT and A5-KO hPSCs towards pancreatic lineage (Fig. 3d). Firstly, we differentiated hPSCs into DE via treatment with Wnt signaling activator CHIR99021 and Activin A with a high concentration. Both WT and A5-KO hPSCs could be efficiently differentiated into DE cells co-expressing SOX17 and FOXA2 (Supplementary Figs. 3d, 4c). Fluorescence-activated cell sorting (FACS) analysis based on DE markers, SOX17, FOXA2, and CXCR4, showed that there were more than 80% DE cells in both samples (Supplementary Figs. 3f, g, 4d, e). We could not observe difference between WT and A5-KO at DE stage, indicating that deficiency of ALKBH5 does not affect DE specification.

Next, we checked the differentiation capacity of WT and A5-KO DE towards PF and found that both WT and A5-KO lines could generate comparable PDX1-positive PF cells (about 80% for both samples) as analyzed by immunostaining and FACS (Supplementary Fig. 3d, h). Thus, ALKBH5 is not required for PF specification.

Subsequently, at the PP stage, we clearly observed that PDX1 and NKX6.1 double-positive cells were less in PPs from A5-KO than in PPs from WT as determined by immunostaining (Fig. 3e; Supplementary Fig. 4c). In addition, FACS results demonstrated that the percentage of PDX1 and NKX6.1 double-positive cells were significantly reduced in A5-KO (Fig. 3f; Supplementary Fig. 4f). RT-qPCR results confirmed that the expression levels of pancreatic progenitor marker genes *PDX1*, *NKX6.1*, *HNF6*, and *SOX9* in A5-KO were lower than those in WT (Fig. 3g; Supplementary Fig. 4g). The protein level of NKX6.1 was also significantly reduced in A5-KO (Fig. 3h and Supplementary Fig. 3i). Next, we checked cell proliferation at PP stage by Ki67 staining, and did not find significant difference between WT and A5-KO lines (Supplementary Fig. 3d). In order to get insights into the transcriptome changes after *ALKBH5* KO, we performed RNA-seq with PP samples. The results demonstrated that 965 and 811 genes were significantly up- and downregulated, respectively, upon *ALKBH5* depletion (Fig. 3i). These downregulated genes included *PDX1*, *SOX9*, *HNF6*, *MNX1*, and *NKX6.1*, all of which were critical for pancreatic lineage specification; while other endodermal lineage marker genes, such as hepatocyte genes *ALB* and *AFP*, were upregulated (Fig. 3j). We checked hepatic marker ALBUMIN (ALB), and observed the increase of ALB^+^ cells in A5-KO at PP stage, indicating that *ALKBH5* deletion may disturb pancreatic specification (Supplementary Fig. 5a). GO analysis identified that these downregulated genes were related to multiple biological processes, including RNA modification, regulation of cell morphogenesis, and pancreas development (Fig. 3k). On the other hand, upregulated genes were enriched for digestive system process and lipid homeostasis, which may be caused by the specification towards other endodermal lineages (Fig. 3k). Collectively, these data demonstrate that ALKBH5 is required for the efficient formation of PDX1 and NKX6.1 double-positive PPs.

### ALKBH5 regulates human pancreatic islet organogenesis

**Fig. 4:**
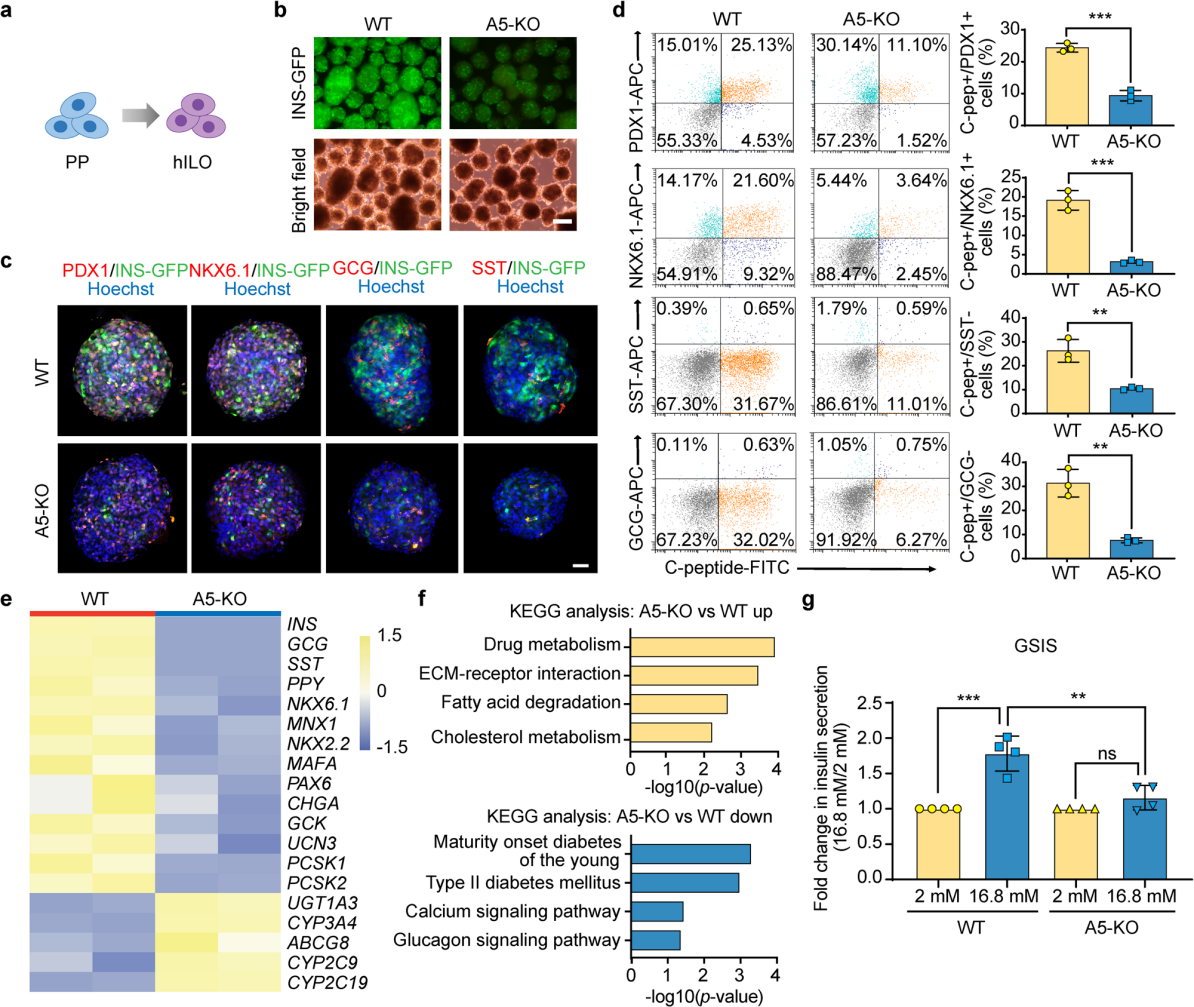
ALKBH5 is required for pancreatic islet organogenesis. **a** A schematic representation of the generation of hILOs from PPs. **b** Representative fluorescence images of INS-GFP-positive pancreatic β-like cells in WT and A5-KO hILOs. Images are representative of four independent replicates. Scale bar, 200 μm. **c** Representative immunofluorescent images of WT and A5-KO hILOs stained for PDX1, NKX6.1, GCG, SST, and nuclei. Scale bar, 100 μm. Images are representative of three independent replicates. **d** Representative flow cytometry plots and the percentage of PDX1^+^C-peptide^+^, NKX6.1^+^C-peptide^+^, SST^-^C-peptide^+^, and GCG^-^C-peptide^+^ cells in WT and A5-KO populations (*n* = 3 biological replicates). **e** Heatmap visualization of marker gene expression. **f** KEGG analysis of upregulated and downregulated genes. *p*-values were calculated by one-tailed hypergeometric test. **g** The GSIS assay of WT and A5-KO hILOs in response to 2 and 16.8 mM glucose respectively (*n* = 4 biological replicates). Data are presented as mean ± s.d. Statistical significance calculated using two-tailed Student’s *t*-test, ns *p* > 0.05, ** *p* < 0.01, *** *p* < 0.001. Source data are provided as a Source Data file.

To check the functions of ALKBH5 in human pancreatic islet organogenesis, we further differentiated WT and A5-KO PPs into hILOs (Fig. 4a). To characterize the in vitro-derived insulin-producing cells, We employed the INS^GFP/W^ reporter cell line in which green fluorescence protein (GFP) expression is mediated by the endogenous insulin promoter^46^. The generation of pancreatic β-like cells (INS-GFP positive cells) was notably inhibited after the ablation of *ALKBH5* (Fig. 4b and Supplementary Fig. 5b). Immunostaining results showed that PDX1^+^, NKX6.1^+^, and INS^+^ cells were significantly reduced at A5-KO-derived hILOs. (Fig. 4c), while the ALB^+^ cells and α-fetoprotein^+^ (AFP^+^) cells were increased in A5-KO-derived-hILOs (Supplementary Fig. 5c). The FACS experiments confirmed the significant reduction of C-peptide^+^ mono-hormonal pancreatic β-like cells in the A5-KO hILOs (Fig. 4d). Next, we performed RNA-seq analysis of hILOs derived from WT and A5-KO hPSCs to further explore the effect of ALKBH5 on human pancreatic islet organogenesis. The RNA-seq result demonstrated that endocrine genes, such as *INS*, *GCG*, *SST*, and *PPY*, were all downregulated in A5-KO-derived hILOs (Fig. 4e). In addition, many pancreatic β cell marker genes, such as *NKX6.1*, *MNX1*, *NKX2.2*, *GCK*, *PCSK1*, and *PCSK2* were also downregulated (Fig. 4e). KEGG analysis demonstrated that downregulated genes were related to maturity onset diabetes of the young, type II diabetes mellitus, calcium signaling pathway, and glucagon signaling pathway (Fig. 4f). Finally, we assessed the function of pancreatic β-like cells by glucose-stimulated insulin-secretion assay (GSIS). Briefly, we stimulated hILOs derived from WT and A5-KO hPSCs with high level of glucose and checked their insulin secretion capacity using ELISA. The function of pancreatic β-like cells was severely impaired in A5-KO-derived hILOs (Fig. 4g).

Taken together, we can conclude that ALKBH5 is responsible for modulating the expression levels of many essential genes that are critical for human pancreatic development and is essential for human pancreatic-lineage specification, islet organogenesis, and pancreatic β cell functions.

### m^6^A demethylation activity of ALKBH5 is required

**Fig. 5:**
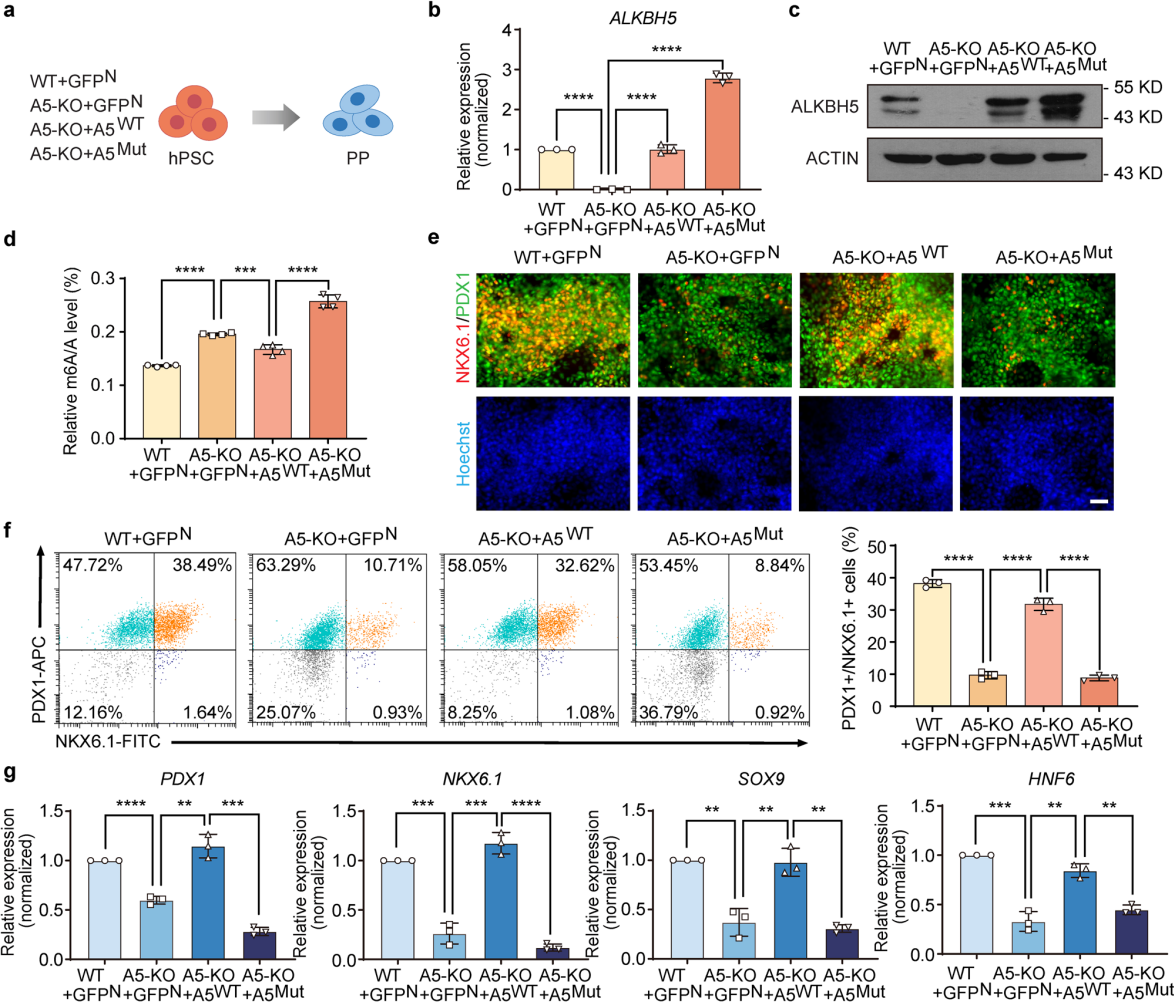
Demethylation activity of ALKBH5 is required for human pancreatic differentiation. **a** A schematic representation of the rescue experiment. WT + GFP^N^, A5-KO + GFP^N^, A5-KO + A5^WT^, and A5-KO + A5^Mut^ hPSC lines were used. **b** RT-qPCR analysis of the expression of *ALKBH5* (*n* = 3 biological replicates). **c** Western blotting analysis of ALKBH5 protein levels in WT + GFP^N^, A5-KO + GFP^N^, A5-KO + A5^WT^, and A5-KO + A5^Mut^ hPSC lines. ACTIN was used as a loading control. Images are representative of three independent replicates. **d** The quantification of m^6^A abundance in WT + GFP^N^, A5-KO + GFP^N^, A5-KO + A5^WT^, and A5-KO + A5^Mut^ hPSC lines by UHPLC-QQQ-MS/MS (*n* = 4 biological replicates). **e** Immunofluorescent staining of PDX1, NKX6.1, and nuclei. Images are representative of three independent replicates. Scale bar, 100 μm. **f** Representative flow cytometry plots and the percentage of PDX1^+^NKX6.1^+^ cells in WT + GFP^N^, A5-KO + GFP^N^, A5-KO + A5^WT^, and A5-KO + A5^Mut^ PP populations (*n* = 3 biological replicates). **g** RT-qPCR analysis of *PDX1*, *NKX6.1*, *SOX9*, and *HNF6* expression (*n* = 3 biological replicates). All data are presented as mean ± s.d. Statistical significance calculated using two-tailed Student’s *t*-test, ** *p* < 0.01, *** *p* < 0.001, **** *p* < 0.0001. Source data are provided as a Source Data file.

To rule out off-target possibility and determine whether enzymatic activity of ALKBH5 is required, we restored *ALKBH5* expression in A5-KO hPSCs for rescue experiments. Previous studies have already shown that the H204A mutation can cause the loss of demethylation activity of ALKBH5^28,47,48^. We generated A5-KO + A5^WT^ and A5-KO + A5^Mut^ hPSC lines by infecting with A5^WT^ and A5^Mut^ lentiviruses. In addition, we infected WT and A5-KO hPSCs with a GFP^N^ lentivirus, and used them as controls. These four hPSC lines were applied for the pancreatic differentiation experiments (Fig. 5a). At first, we checked the *ALKBH5* expression level in these cell lines, and observed the restoration of *ALKBH5* expression in A5-KO + A5^WT^ and A5-KO + A5^Mut^ hPSC lines (Fig. 5b). Western blotting results also showed the recovery of ALKBH5 on the protein level (Fig. 5c). By UHPLC-QQQ-MS/MS, we confirmed that the m^6^A level was also rescued after ALKBH5 restoration (Fig. 5d). Next, we differentiated these hPSC lines into pancreatic lineage as previously described. We checked the formation of PPs by immunostaining and FACS, and found that A5^WT^ could recover the differentiation capacity of A5-KO hPSCs, suggesting that the deficiency of pancreatic differentiation capacity of A5-KO hPSCs was caused by *ALKBH5* KO rather than the off-target effects (Fig. 5e, f). On the other hand, A5^Mut^ failed to rescue the defects in pancreatic differentiation caused by *ALKBH5* deficiency, indicating that enzymatic activity of ALKBH5 is required for pancreatic differentiation (Fig. 5e, f). The RT-qPCR results further supported the phenotypic observations. The gene expression levels of pancreatic progenitor cell markers, including *PDX1*, *NKX6.1*, *SOX9*, and *HNF6* significantly increased after A5^WT^ expression in the A5-KO cells (Fig. 5g). Together, these results indicate that ALKBH5, along with an intact demethylase activity, is necessary for hPSC-to-PP specification.

### Identification of potential targets of ALKBH5

**Fig. 6:**
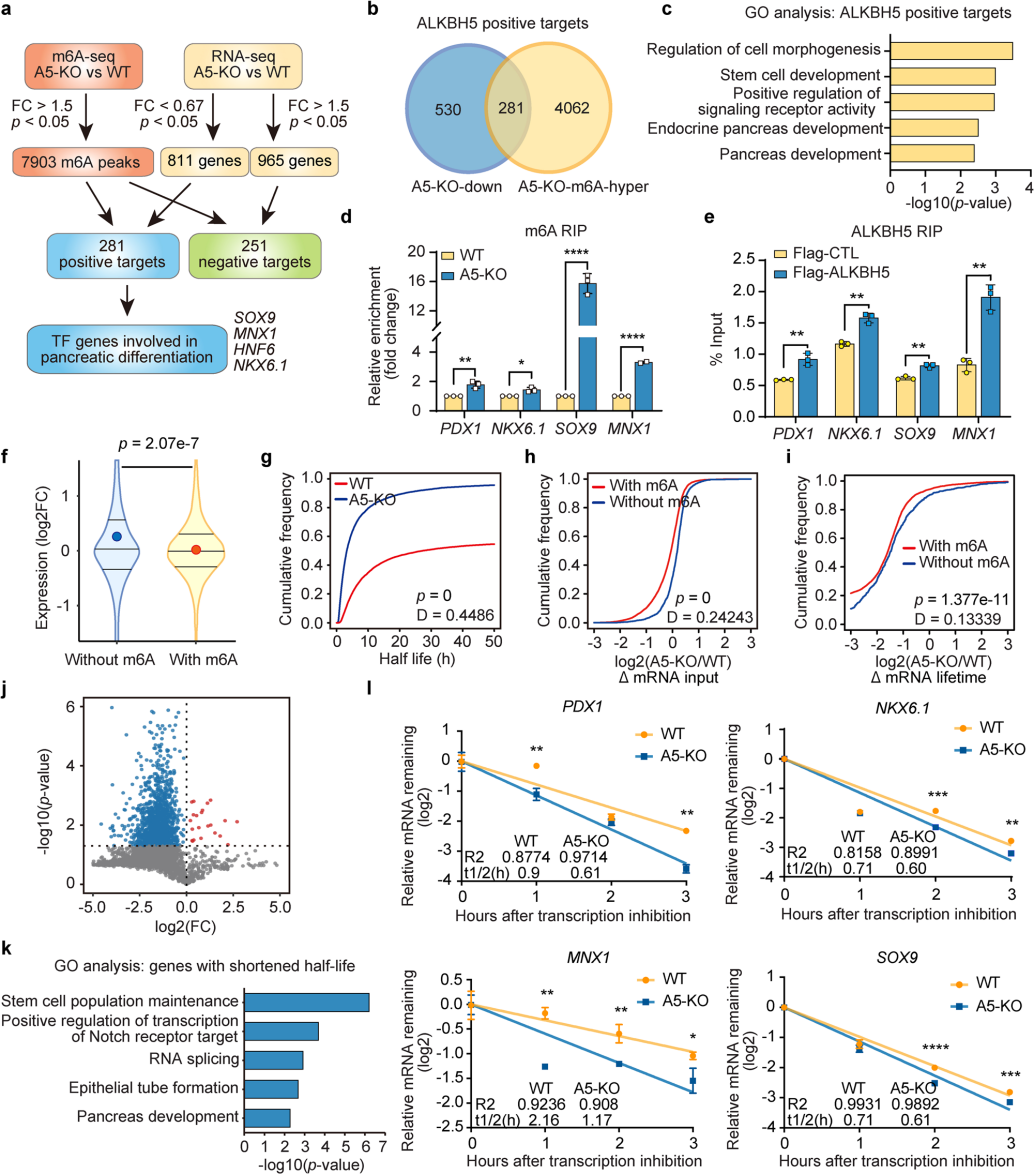
Molecular mechanism of ALKBH5 in regulating human pancreatic lineage specification. **a** Flowchart of ALKBH5 downstream target screening. **b** Venn diagram showing the integrative analysis to identify potential ALKBH5 targets. A5-KO-down: significantly downregulated genes in A5-KO PPs. A5-KO-m^6^A-hyper: genes with significantly higher m^6^A abundance in A5-KO PPs. **c** GO categories of the positive targets of ALKBH5, *p*-values were calculated by one-tailed hypergeometric test. **d** RIP-qPCR showing the m^6^A enrichment increased on indicated mRNA transcripts in A5-KO PPs compared with WT PPs (*n* = 3 biological replicates). **e** RIP-qPCR analysis of the indicated mRNA transcripts bound with ALKBH5 in PPs (*n* = 3 biological replicates). **f** Violin plots showing expression changes between A5-KO and WT PPs for methylated (with m^6^A) and not-methylated (without m^6^A) transcripts. The upper and lower quartiles and the median are indicated for each group. Dots, the average value of fold changes. *p*-value was calculated using two-tailed Wilcoxon test. **g** Cumulative distributions of global transcript half-life changes in WT and A5-KO PPs. **h** Cumulative distributions of mRNA input changes (A5-KO/WT, log_2_FC) of methylated (with m^6^A) and not-methylated (without m^6^A) transcripts. **i** Cumulative distributions of mRNA lifetime changes (A5-KO/WT, log_2_FC) of methylated (with m^6^A) and not-methylated (without m^6^A) transcripts. **j** Volcano plot of transcripts with significant half-life change in A5-KO PPs compared to WT PPs. Red dots: transcripts with increased half-life; blue dots: transcripts with shortened half-life. **k** GO categories of genes with shortened half-life, *p*-values were calculated by one-tailed hypergeometric test. **l** The decay curves for *PDX1*, *NKX6.1*, *MNX1*, and *SOX9* in WT and A5-KO PPs (*n* = 3 biological replicates). All data are presented as mean ± s.d. Statistical significance calculated using two-tailed Student’s *t*-test, * *p* < 0.05, ** *p* < 0.01, *** *p* < 0.001, **** *p* < 0.0001. Source data are provided as a Source Data file.

To decipher the underlying mechanisms of ALKBH5 involved in human pancreatic differentiation, we conducted m^6^A-seq for samples at PP stage, starting from which step we could obviously detect the phenotypic differences between WT and A5-KO. The m^6^A-seq data demonstrated that a vast majority of m^6^A peaks were distributed in the CDS and 3′UTR of transcripts (Supplementary Fig. 6a–c). Particularly, the m^6^A peak density in 3′UTR and near stop codon increased after *ALKBH5* KO, which was consistent with its functional role as an m^6^A eraser. Next, we took integrative analysis of m^6^A-seq and input RNA-seq data (Fig. 6a). We found that 7903 m^6^A peaks located on 4343 genes revealed hyper methylation in A5-KO versus WT PPs (Fig. 6a, b). Especially, 281 genes among them were downregulated according to the RNA-seq results, which might imply the role of m^6^A modification for mediating mRNA decay (Fig. 6b). GO analysis suggested that these potential targets were involved in the regulation of cell morphogenesis, stem cell development, and pancreas development (Fig. 6c).

Interestingly, several transcription factors that are important for pancreatic lineage specification, such as *MNX1*, SO*X9*, *HNF6*, and *NKX6.1*, were identified as candidate targets of ALKBH5 (Fig. 6a). We analyzed other published pancreatic differentiation datasets, and found that the expression of *ALKBH5* was positively correlated with pancreatic progenitor maker genes *PDX1*, *NKX6.1*, *SOX9*, *MNX1*, while *HNF6* only showing a weakly and insignificantly positive correlation (Supplementary Fig. 6d). Next, we detected the m^6^A enrichment on the mRNAs of these five transcription factors. The m^6^A-RIP-qPCR results confirmed that the m^6^A modification levels of *PDX1*, *NKX6.1*, *MNX1*, and *SOX9* were indeed upregulated after *ALKBH5* deletion (Fig. 6d and Supplementary Fig. 6e, f). Further, the ALKBH5-RIP-qPCR experiment validated that ALKBH5 could bind with transcripts of *PDX1*, *NKX6.1*, *MNX1*, and *SOX9* (Fig. 6e). Taken together, we have identified key pancreatic differentiation maker genes as ALKBH5 targets, including *PDX1*, *NKX6.1*, *MNX1*, and *SOX9*.

### m^6^A-mediated mRNA metabolism regulates differentiation

Next, we wanted to uncover how ALKBH5 regulated its targets during pancreatic differentiation. As the m^6^A modification has been implicated in the control of mRNA metabolism, including stability and degradation^30^, we firstly analyzed the RNA-seq data of PP-WT and PP-A5-KO, and found that mRNA transcripts without m^6^A modification were generally more stable than mRNAs with m^6^A modification (Fig. 6f). Therefore, we explored the mRNA degradation in WT and A5-KO PPs. WT and A5-KO PPs were treated with transcription inhibitor actinomycin D (Act D), and collected at 0, 1, 2, and 3 h, respectively. Then we evaluated the half-life of mRNAs by RNA-seq. Interestingly, we observed reduced global mRNA stability in A5-KO cells (median of mRNA half-lives in A5-KO cells: 3.11 h; median of mRNA half-lives in WT cells: 7.81 h, *p* = 0, Mann–Whitney *U*-test) (Fig. 6g). A further analysis showed that mRNAs without m^6^A modification were generally more stable compared to those with m^6^A modification transcripts in A5-KO samples (*p* = 0, Mann–Whitney *U*-test) (Fig. 6h). Moreover, *ALKBH5* deletion led to shorter (~38% in average) lifetimes of mRNAs harboring m^6^A modification in comparison with mRNAs without m^6^A modification (*p* = 1.377e^−11^, Mann–Whitney *U*-test) (Fig. 6i). Notably, 1555 transcripts showed significantly decreased half-lives after *ALKBH5* deletion, whereas only a few (*n* = 22) transcripts had increased half-lives (Fig. 6j). GO analysis demonstrated that genes with shortened half-life were enriched for stem cell population maintenance, Notch signaling, and pancreatic development (Fig. 6k). Among these transcripts, we further confirmed that mRNA stabilities of key pancreatic differentiation marker genes, including *PDX1*, *NKX6.1*, *MNX1*, and *SOX9*, indeed significantly decreased in A5-KO cells (Fig. 6l and Supplementary Fig. 6g).

**Fig. 7:**
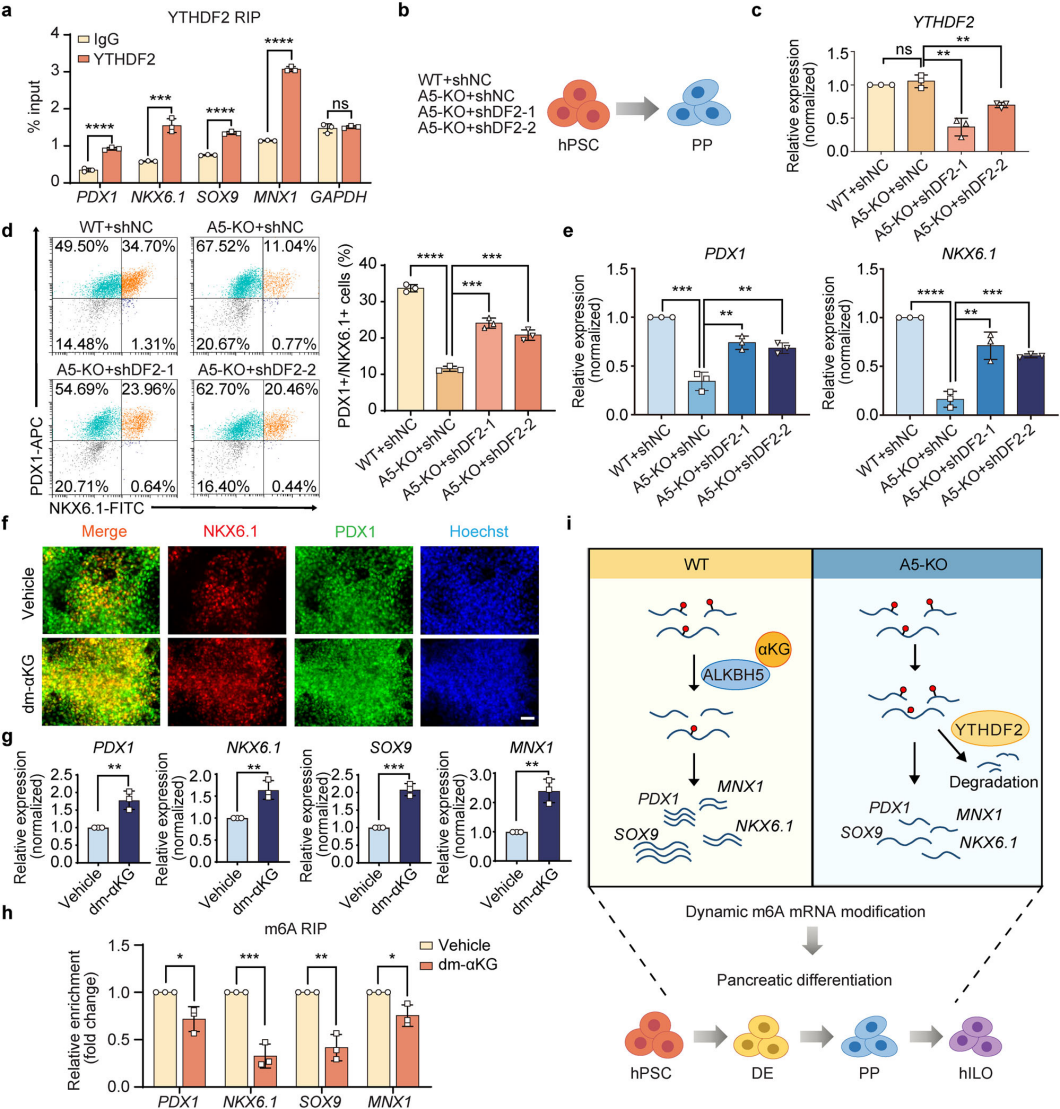
YTHDF2 knock-down rescues and dm-αKG promotes human pancreatic differentiation. **a** RIP-qPCR analysis of the indicated mRNA transcripts bound with YTHDF2 in PPs (*n* = 3 biological replicates). **b** A schematic representation of the generation of PPs from hPSCs. WT + shNC, A5-KO + shNC, A5-KO + shDF2-1, and A5-KO + hDF2-2 hPSC lines were used. **c** RT-qPCR analysis of the expression of *YTHDF2* (*n* = 3 biological replicates). **d** Representative flow cytometry plots and the percentage of PDX1^+^NKX6.1^+^ cells in WT + shNC, A5-KO + shNC, and A5-KO + shDF2 PPs (*n* = 3 biological replicates). **e** RT-qPCR analysis of *PDX1* and *NKX6.1* expression (*n* = 3 biological replicates). **f** Immunofluorescent staining of PPs for PDX1, NKX6.1, and nuclei. Images are representative of three independent replicates. Scale bar, 100 μm. **g** RT-qPCR analysis of *PDX1*, *NKX6.1*, *SOX9*, and *MNX1* expression (*n* = 3 biological replicates). **h** RIP-qPCR showing the m^6^A enrichment decreased on indicated mRNA transcripts in PP populations treated with dm-αKG (*n* = 3 biological replicates). **i** A schematic summary of functions and molecular mechanisms of ALKBH5 during human pancreatic specification and islet organogenesis. All data are presented as mean ± s.d. Statistical significance calculated using two-tailed Student’s *t*-test, ns *p* > 0.05, * *p* < 0.05, ** *p* < 0.01, *** *p* < 0.001, **** *p* < 0.0001. Source data are provided as a Source Data file.

The effects of m^6^A modification are mediated by cell-type-specific m^6^A reader proteins. For example, YTHDF1 and YTHDF3 promote translation, and IGF2BP1-3 can stabilize m^6^A methylated transcripts. Notably, YTHDF2 has been known to specifically recognize m^6^A and trigger the rapid degradation of m^6^A-containing mRNAs^30,49^. We found that the expression of *YTHDF2* significantly increased in PP stage during differentiation, and was much higher than those of other readers in PPs (Supplementary Fig. 7a–c). Based on these facts and observations, we thought that YTHDF2 played the major role to perform the function, and thus tested the effects of YTHDF2. First, we applied YTHDF2-RIP-qPCR, and validated the binding of *PDX1*, *NKX6.1*, *MNX1*, and *SOX9* transcripts with YTHDF2 (Fig. 7a). Then, we knocked down *YTHDF2* by shRNAs (shDF2-1 and shDF2-2) and evaluated the effects on pancreatic differentiation (Fig. 7b, c). As shown in Fig. 7d, shDF2s could partially rescue the defect of pancreatic differentiation caused by A5-KO (Supplementary Fig. 8a). In addition, shDF2 partially restored the expressions of *PDX1*, *NKX6.1*, *MNX1*, and *SOX9* (Fig. 7e and Supplementary Fig. 8b).

Collectively, these results support that ALKBH5 regulates human pancreatic differentiation through manipulating mRNA m^6^A modification and stabilities of critical genes involved in human pancreatic differentiation and development.

### α-ketoglutarate-ALKBH5-m^6^A axis promotes differentiation

Based on our observations above, it would be very attractive to improve human pancreatic differentiation through modulating m^6^A modification. However, to date, specific and potent activators of ALKBH5 remain very limited. α-ketoglutarate (αKG) is the cofactor of ALKBH5, and therefore we tested the effects of αKG on human pancreatic differentiation. Notably, we observed that cell-permeable dimethyl-αKG (dm-αKG) significantly increased the percentage of PDX1 and NKX6.1 double-positive PPs (Fig. 7f and Supplementary Fig. 8c). RT-qPCR showed that dm-αKG upregulated the expression of *PDX1*, *NKX6.1*, *SOX9*, and *MNX1* (Fig. 7g). Consistently, the m^6^A levels of *PDX1, NKX6.1, SOX9*, and *MNX1* mRNAs decreased after dm-αKG administration (Fig. 7h). Therefore, αKG-ALKBH5-m^6^A axis can be harnessed to promote human pancreatic differentiation and will facilitate its translational applications.

## Discussion

During cell-fate transitions, transcriptome changes are dynamically regulated and highly coordinated at multiple levels. Besides epigenetic modulations, such as DNA and histone methylation, mRNA m^6^A methylation has recently been recognized as an important layer of controls over cellular differentiation and organ development^34^. In this report, using hPSC-based pancreatic differentiation platform, we investigated mRNA m^6^A dynamics during pancreatic lineage specification and further explored the roles of m^6^A modification and its demethylase ALKBH5 in pancreatic differentiation.

For technical issues, it is challenging to collect large amounts of samples for m^6^A-seq, and there is so far no report for describing mRNA m^6^A dynamics during pancreatic lineage specification^50^. Based on the directed differentiation platform of hPSCs to hILOs, we found that the pancreatic lineage progression was accompanied by changes in m^6^A modification on numerous transcripts. This study of mRNA m^6^A dynamics during pancreatic differentiation presents a valuable resource for further exploration.

Using CRISPR-based precise genome editing tool, we generated *ALKBH5* KO hPSC lines. Then, we investigated the roles of ALKBH5 during human pancreatic differentiation in more details. Unexpectedly, we found that ALKBH5 played critical roles on pancreatic lineage specification. Previously, *Alkbh5* KO mice were reported to be normal only with impaired fertility^28,51^. and no abnormal phenotype was reported on pancreatic development. Our study supports that hPSCs can offer a unique opportunity for investigating human disease phenotypes that are not readily recapitulated in model organisms.

Mechanistically, we found that m^6^A modifications of many essential transcription factors like *PDX1*, *NKX6.1*, *SOX9*, and *MNX1* involved in pancreatic development were altered after *ALKBH5* depletion (Fig. 7i). m^6^A modification influences mRNA metabolism in almost every step of its lifecycle, including mRNA splicing, export, stability, and translation efficiency^23,35^. We further identified *PDX1*, *NKX6.1*, *MNX1*, and *SOX9* as the direct targets of ALKBH5, and showed that m^6^A modification could regulate the stability of these mRNA transcripts through YTHDF2-mediated mRNA decay pathway. In addition, we found that ALKBH5 cofactor αKG could decrease the m^6^A modification of these mRNA transcripts and significantly promote human pancreatic differentiation. In the future, more potent molecules can be developed for specifically modulating ALKBH5 and YTHDF2, which will facilitate better understanding of the molecular mechanisms of m^6^A modification.

In sum, our study demonstrates that m^6^A mRNA modification is highly dynamic during pancreatic differentiation. Accordingly, ALKBH5 plays important regulatory roles in regulating essential gene expression in pancreatic progenitors and islet endocrine cells. Therefore, harnessing mRNA m^6^A regulation would provide strategies for controlling human pancreatic differentiation and developing effective approaches for generating large amounts of functional islets for the study of pancreatic biology and treatment of various metabolic diseases.

## Methods

### Statement

This work complies with all relevant ethical regulations and was reviewed by the Internal Review Committee of Zhejiang University.

### Plasmid construction

The wild-type *ALKBH5*-CDS was cloned into the pSin-BSD vector to generate pSin-*ALKBH5*. For pSin-*ALKBH5*^Mut^ plasmid, *ALKBH5 H204A* mutation^28,47,48^ was introduced using the Gibson Assembly kit (New England Biolabs). For pSin-*GFP*^N^ plasmid, the sequence of *GFP* N-terminal domain was linked with pSin-BSD backbone by using Gibson Assembly kit. For shRNA plasmid construction, single-strand DNA was synthesized from Sangon Biotech. After annealing, the insert DNA was ligated with pLKO.1 vector. All vectors were checked by Sanger sequencing.

### Cell culture

Human embryonic kidney 293T (HEK293T) cells were cultured in Dulbecco’s Eagle Medium (DMEM) (Life Technologies), 10% fetal bovine serum (FBS) (Gibco), and 1× Penicillin/Streptomycin (Life Technologies). HEK293T cells were from ATCC (CRL-3216).

Undifferentiated hPSCs were cultured in hPSC medium: DMEM/F12 (Life Technologies), 20% KnockOut Serum Replacement (KSR) (Life Technologies), 1× Non-Essential Amino Acids (NEAA) (Life Technologies), 0.055 mM 2-mercaptoethanol (Sigma), 1× Penicillin/Streptomycin, and 10 ng ml^−1^ bFGF (Peprotech). hPSCs were maintained on CF1 feeder cells at 37 °C and 5% CO_2_. hPSCs were isolated by Accutase (Life Technologies) as 1:3 to 1:6 every 3–6 days. 0.5 μM thiazovivin (TargetMol) were used during the first 24 h when passaging or thawing cells. MEL1 INS^GFP/W^ hESC line^46^ was a kind gift from Drs. E. G. Stanley and Andrew Elefanty. Mycoplasma contamination was routinely detected using *TransDetect* PCR Mycoplasma Detection Kit (TransGen Biotech).

### Generation of *ALKBH5* knockout hPSC lines

CRISPR-Cpf1 crRNAs were designed using an online software (http://chopchop.cbu.uib.no) to target the first exon of *ALKBH5* locus. Two most efficient crRNAs (cr1: GAGTGGGTGCACCAGCTGGTGATCCAAA; cr2: TTTAGCGACTCTGCGCTGTGCTTCGGCT) were used to produce indel mutations. Plasmids were transfected into hPSCs according to our previous protocol^13^. All plasmids (pcDNA3.1-hLbCpf1 (Addgene, Plasmid #31938), pCpfcr-cr1, and pCpfcr-cr2) were extracted by ZymoPURE Plasmid Maxiprep Kit (ZYMO). 1 × 10^6^ cells were resuspended with plasmid mixture and electroporation solution (Human Stem Cell Nucleofector Kit 1, Lonza) followed by electroporation. After electroporation, cells were cultured in one well of six-well plate for 2–3 days. Subsequently, 500–2000 cells were passaged onto 10 cm dishes and cultured for about one week. hPSC colonies were picked, expanded, genotyped, and banked for further studies. Primer sequences for genotyping are listed in Supplementary Table 3.

### Human pancreatic differentiation from hPSCs

hPSCs were differentiated into pancreatic lineage by a previously described protocol^4^. hPSCs were seeded into 12-well plates at a density of 5 × 10^5^ per well and the differentiation was initiated 48 h after seeding. hPSCs were quickly washed by DPBS (Life Technologies) and exposed to differentiation media. Here is the detailed media: Day 1: RPMI (Life Technologies), 1× Penicillin/Streptomycin, 100 ng ml^−1^ activin A, and 3 μM CHIR99021 (TargetMol). Day 2: RPMI, 0.2% FBS, 1× Penicillin/Streptomycin, and 100 ng ml^−1^ activin A. Day 3: RPMI, 2% FBS, 1× Penicillin/Streptomycin, and 100 ng ml^−1^ activin A. Day 4–6: RPMI, 0.5× B27 (Gibco), 0.5× N2 (Gibco), 0.05% BSA (Yeasen), 1× Penicillin/Streptomycin, and 50 ng ml^−1^ KGF (Peprotech). Day 7–8: DMEM, 1× B27, 0.05% BSA, 1× Penicillin/Streptomycin, 0.25 mM vitamin C (Sigma-Aldrich), 50 ng ml^−1^ KGF, 0.1 μM LDN-193189 (Tocris), 0.1 μM GDC-0449 (Selleck), and 2 μM retinoic acid (Sigma). Day 9–14: DMEM, 1× B27, 0.05% BSA, 1× Penicillin/Streptomycin, 0.25 mM vitamin C, 0.1 μM LDN-193189, and 50 ng ml^−1^ EGF (Peprotech). Then, cells were suspended to form aggregates, and differentiated into hILO stage using medium modified from our previous protocol^52^. Day 15–22 (R6 medium): DMEM, 1:50 B27, 0.05% BSA, 1× Penicillin/Streptomycin, 10 μM zinc sulfate (Sigma), 10 μg ml^−1^ heparin (Sigma), 10 μM 616452 (TargetMol), 1 μM T3 (Sigma), 0.1 μM LDN-193189, 0.1 μM compound E (MedChem Express), and 0.25 mM vitamin C. Day 23–30 (R7 medium): DMEM, 1× B27, 0.05% BSA, 1× Penicillin/Streptomycin, 10 μM zinc sulfate, 10 μg ml^−1^ heparin, 10 μM 616452, 1 μM T3, 1 mM *N*-acetyl cysteine (Sigma), 1 μM trolox (Sigma), and 0.25 mM vitamin C.

### Rescue experiments

Lentiviruses were produced in HEK293T cells using Lipofectamine™ 3000 Transfection Reagent (Invitrogen). For lentiviral infection, 60–70% confluent hPSCs were incubated with viruses for 4 h. Then cells were cultured in hPSC medium for about one week, and 10 μg ml^−1^ blastmycin (YEASEN) was used to select positive cells from day 3.

### Generation of *YTHDF2* knockdown hPSC lines

hPSCs were transduced with lentiviruses expressing shRNAs (shYTHDF2-1: 5′-GAACGTCAAGGTCGTGGGAAA-3′, shYTHDF2-2: 5’-TACTGATTAAGTCAGGATTAA-3′ and shNC: 5′-CAACAAGATGAAGAGCACCAA-3′). Selection of efficiently transduced cells was achieved by treatment with puromycin (1 μg ml^−1^ final concentration) for about 3 days.

### Immunostaining

Cells were stained according to our previous protocol^13^. In detail, cells were fixed with 4% paraformaldehyde (PFA) for 10–15 min at room temperature (RT), washed with PBST buffer (PBS + 0.3% Triton X-100) for three times, blocked in blocking buffer (PBST + 5% BSA) for 1 h at RT followed by incubated with the primary antibody at 4 °C overnight. Then, secondary antibodies were used at 1:2000 dilution and incubated for 1 h at RT. Detailed primary and secondary antibodies are listed in Supplementary Table 1. Finally, cells were stained with Hoechst at 1:5000 dilution to mark nuclei.

### Western blotting

Lysis buffer (Beyotime) supplemented with 1% PMSF (Beyotime) was used to extract total protein from cells. Cell extracts were centrifuged at 12,000 × *g* for 15 min and the supernatants were collected. Cell lysates were resolved on 10% acrylamide gradient SDS–PAGE gels and transferred to PVDF membranes (Millipore). The membranes were blocked with 5% non-fat milk in Tris-buffered saline containing 0.1% Tween 20 for 1 h, incubated with primary and secondary antibodies, and detected by immunoblotting with the High Sensitivity ECL Chemiluminescence Detection Kit (Vazyme) or Super ECL Detection Reagent (Yeasen Biotech). Primary and secondary antibodies are outlined in Supplementary Table 1.

### Flow cytometry

Cells were dissociated into single cells using Accutase and washed with PBS buffer. Then, cells were fixed by 4% PFA for 30 min and washed by PBST for 3–5 times followed by centrifuged at 200 × *g*, 5 min per time. Thereafter, cells were blocked with blocking buffer and incubated with primary antibodies at 4 °C overnight. After washing with PBST for 3 times, cells were incubated in secondary antibodies at RT for 1 h. All the antibodies are detailed in Supplementary Table 1. FACS data were acquired by Beckman CytoFlex (Beckman Culture) and analyzed by CytExpert software.

### Real-time qPCR

Total RNA was extracted and purified using Quick-RNA MiniPrep Kit (ZYMO) and converted into cDNA using PrimeScript RT Master Mix (Takara). RT-qPCR was performed using TB Green Premix Ex Taq II Kit (Takara) on CFX Connect Real-Time system (Bio-Rad). Primer sequences are listed in Supplementary Table 2.

### RNA stability assays and mRNA stability profiling

WT and A5-KO PPs were treated with actinomycin D (Sigma) at a final concentration of 1 μg ml^−1^ and collected at indicated time points. Total RNA was extracted by Fast Pure Cell Total RNA Isolation Kit (Vazyme) and analyzed by RT-qPCR and RNA-seq. The half-life of mRNA was calculated according to a previously published paper^53^. After inhibiting transcription by actinomycin D treatment, the change of mRNA concentration at a given time (d*C/*d*t*) is proportional to both the rate constant for decay (**k**_decay_) and mRNA concentration (*C*) as shown in the following equation:

1 $${{{{{\rm{d}}}}}}C/{{{{{{\rm{d}}}}}}t}=-{{{{{{\bf{k}}}}}}}_{{{{{{\rm{decay}}}}}}}C$$

The minus symbol indicates that the mRNA is being degraded rather than synthesized. This relationship leads to the derivation of the equation:

2 $${{{{{\rm{ln}}}}}}(C/{C}_{0})=-{{{{{{\bf{k}}}}}}}_{{{{{{\rm{decay}}}}}}}t$$

When 50% of mRNA is decayed (*C/C*_*0*_ = 1/2), the mRNA half-life (*t*_*1/2*_) can be calculated by the equation:

3 $${{{{{\rm{ln}}}}}}(1/2)=-{{{{{{\bf{k}}}}}}}_{{{{{{\rm{decay}}}}}}}{t}_{1/2}$$

from where:

4 $${t}_{1/2}={{{{{\rm{ln}}}}}}2/{{{{{{\bf{k}}}}}}}_{{{{{{\rm{decay}}}}}}}$$

For RNA-seq data, cDNA library construction and high-throughput sequencing were performed by Novogene, raw RNA-seq data were trimmed by fastp (v0.20.1) to remove adapter sequence and reads with low sequencing quality, paired-end sequencing was carried out with the Illumina HiSeq 2500 with paired-end 150 bp read length. Clean reads were aligned to the human genome (GRCh38) using HISAT2 (v2.1.0) with the default parameter settings. Transcript assembly was performed by stringtie (v2.0)^54^ and expression of transcripts sharing each gene_id was quantified as transcripts Per Million (TPM). TPM was converted to attomoles by linear fitting of the RNA ERCC spike-ins. The degradation rate of RNA and the mRNA half-life were calculated according to the aforementioned formula. The final half-life was calculated by using the average value of 0, 1, 2, and 3 h.

### Glucose-stimulated insulin-secretion (GSIS)

hILOs were washed twice with 1 ml KRBH buffer (128 mM NaCl, 5 mM KCl, 2.7 mM CaCl_2,_ 1.2 mM MgCl_2_, 1 mM Na_2_HPO_4_, 1.2 mM KH_2_PO_4_, 5 mM NaHCO_3_, 10 mM HEPES, 0.1% BSA). Then, clusters were preincubated in 3 ml KRBH with 2 mM glucose (low glucose KRBH) for 1 h to remove residual insulin. During incubation, all tube lids were left open for air exchange. Clusters were washed twice with KRBH buffer followed by incubated in 1 ml low glucose KRBH for 1 h. After incubation, 200 μl of the supernatant were collected for ELISA analysis (low glucose sample). Then, clusters were washed twice in KRBH and then incubated in KRBH with 16.8 mM glucose (high glucose KRBH) for 1 h, and 200 μl of supernatant were collected after incubation (high glucose sample). Finally, clusters were dispersed into single cells using Trypsin for cell counting. Supernatant samples were detected by Human insulin immunoassay kit (EZassay).

### Quantification of m^6^A in RNA by UHPLC-QQQ-MS/MS

Polyadenylated RNAs were purified from total RNA using a GenElute mRNA Miniprep Kit (Sigma), and 200 ng of them were digested by nuclease P1 (1U) (Wako) in 30 μl reaction buffer containing 20 mM of CH_3_COONH_4_ (pH 5.3) at 42 °C for 2 h, followed by additions of 1 μl shrimp alkaline phosphatase (NEB) together with 3.5 μl 10× CutSmart buffer (NEB) and incubated at 37 °C for another 2 h. The samples were diluted to 60–70 μl with DEPC-treated water and filtered (0.22 μm pore size, Millipore), and 10 μl of the solution were injected into UHPLC-QQQ-MS/MS. The nucleosides were quantified by AB Sciex Qtrap 6500+ using the nucleoside to base ion mass transitions of 282.1–150.1 (m^6^A), and 268.2–136.1 (A). Quantification was performed in comparison with the standard curve obtained from pure nucleoside standards running on the same batch of samples. The ratio of m^6^A to A was calculated based on the calibration curves.

### m^6^A-seq

For samples from hPSC, DE, PP, and hILO stages, total RNAs were extracted with TRIzol™ Reagent (Invitrogen), and mRNAs were further enriched by GenElute mRNA Miniprep Kit (Sigma). m^6^A-seq experiments were performed following previously published protocol^55^. Briefly, mRNAs were fragmented into about 100-nt fragments and immunoprecipitated (IP) with 5 μg m^6^A antibody (SYSY, Cat#202003), both input and IP products were subjected to library construction (Illumina). For samples from WT and A5-KO PPs, total RNAs (2–10 μg) were extracted, fragmented, and subjected to immunoprecipitation directly due to limited materials. rRNAs in input fragments were first depleted by rRNA Depletion Kit (NEB), then both input and IP fragments were subjected to library preparation using SMARTer® Stranded Total RNA-Seq Kit v2 (Takara)^44^. All prepared libraries were then sequenced on Illumina Hiseq X10 system with paired-end 150 bp read length.

### m^6^A-RIP-qPCR

Total RNAs were extracted, fragmented and immunoprecipitated by m^6^A-antibody. Both input and IP fragments were reversely transcribed (Takara) and RT-qPCR experiments were performed using iTaq Universal SYBR Green Supermix (Bio-Rad) on Bio-Rad CFX96 Connect Real-Time system. GAPDH was chosen as negative control for calculation of m^6^A enrichment level as following: the expression levels of selected genes in both input and m^6^A-IP samples were first normalized to GAPDH, and the m^6^A enrichment was calculated as m^6^A IP/input. Primer sequences are listed in Supplementary Table 2.

### ALKBH5- and YTHDF2-RIP-qPCR

About 4 × 10^7^ PPs were re-suspended with 3 packed cell volume of lysis buffer (150 mM KCl, 2 mM EDTA, 0.5% NP-40, 0.5 mM DTT, 50 mM Hepes pH 7.5, 1:100 protease inhibitor cocktail, 200 U ml^−1^ RRI (Takara)), pipetted up and down several times and incubated on ice for 15 min and treated with ultrasonic for 1 min then centrifuge at 14,000 × *g* for 30 min at 4 °C, the supernatant was passed through a 0.22 μm membrane syringe filter, saved 1/50 as input and mixed with 1 ml trizol for RNA extraction. The rest cell lysate was used for endogenous YTHDF2-IP as follows: the cell lysate was incubated with 5 μg anti-YTHDF2 rabbit polyclonal antibody (Proteintech, Cat#24744-1-AP) and IgG as negative control at 4 °C overnight, 30 μl protein A beads were washed with binding buffer (300 mM KCl, 1.5 mM MgCl_2_, 0.05% NP-40, 2 mM EDTA, 0.5 mM DTT, 50 mM Hepes, pH 7.5) for three times, resuspended in 500 μl binding buffer (1% protease inhibitor cocktail and 200 U ml^−1^ RRI added) and incubated with cell lysate-antibody mixture at 4 °C for another 4 h, protein A beads were collected with magnetic stand and washed with binding buffer (1% protease inhibitor cocktail and 200 U ml^−1^ RRI added) for three times and then mixed with 500 μl trizol to get RNA and saved as IP. Equal amount of input and IP products were reversed transcribed with random hexamer and subjected to qPCR as described using primers listed in Supplementary Table 2. The enrichment level of selected targets was calculated as 2^-[Ct(IP)-Ct(input)]. For RIP of ALKBH5, PPs expressing flag tagged ALKBH5 were used, and 30 μl anti-flag magnetic beads (Sigma) were used for the enrichment of ALKBH5 following the same procedures.

### m^6^A-seq analysis

All libraries were sequenced on Illumina Hiseq X10 with paired-end 150 bp read length. The deep sequencing data were first trimmed by fastp (v0.20.1)^56^ to remove adapter sequence and reads with low sequencing quality, clean data were then aligned to human reference genome version 38 (GRCh38) using HISAT2 (v2.1.0)^57^ with parameters:–rna-strandness = FR -k 1. The m^6^A peaks were called using R package exomePeak2 (IP/input ≥ 1.5, *p* value < 10^−5^) from the Bioconductor project [http://www.bioconductor.org/]. The longest isoform was retained if a gene has more than one isoform. The differential m^6^A peaks were calculated using R package exomePeak2 with parameters: |log_2_ FC| ≥ 1, adjusted *p* value < 0.05. Motif enrichment was done using HOMER (v4.11)^58^ selecting a motif length of 5, 6, and 7 nucleotides.

### RNA-seq analysis

Total RNA was isolated using Quick-RNA MiniPrep Kit (ZYMO). cDNA library construction and high-throughput sequencing were performed by Novogene, raw RNA-seq data were trimmed by fastp (v0.20.1) to remove adapter sequence and reads with low sequencing quality, paired-end sequencing was carried out with the Illumina HiSeq 2500 with paired-end 150 bp read length. Clean reads were aligned to the human genome (GRCh38) using HISAT2 (v2.1.0) with the default parameter settings. Transcript assembly was performed by stringtie (v2.0)^54^ and expression of transcripts sharing each gene_id was quantified as transcripts Per Million (TPM). The differential expression analysis was performed by R package DESeq2^59^ from the Bioconductor project, the threshold of significantly differentially expressed genes: FC > 1.5 or <0.67 and adjusted *p* value < 0.05. Heatmaps were generated by R package pheatmap from CRAN [https://cran.r-project.org/].

### GO and KEGG analysis

The visualization of GO and KEGG enrichment analysis was used R package clusterProfiler^60^ from the Bioconductor project, and adjusted *p*-value < 0.05 was considered as statistically significant.

### Inheritance and origin analyses of m^6^A peaks

For m^6^A peak’s inheritance and origin analyses in hPSCs to hILOs, we defined the m^6^A peaks with location overlap >50% between a stage and the previous stage as m^6^A peaks inherited from the previous stage, and the remaining peaks in this stage were stage-specific peaks. The m^6^A peak would be used for analysis when the TPM of this m^6^A-tagged transcript >5. The origin analysis is similar to the inheritance analysis. We defined m^6^A peaks with location overlap >50% between a stage and hPSCs as the m^6^A peaks originating from hPSCs. For the m^6^A peaks originating from DE, in addition to doing the same analysis, it was also necessary to remove the m^6^A peaks originating from hPSCs. Similarly, for PP stage, it was needed to remove the m^6^A peaks originating from hPSCs and DE.

### Correlation analysis between gene expression and m^6^A modification

For calculating the relative m^6^A level for each gene, we used the R package DESeq2^59^ to compare the read counts of genes between IP and input samples on a transcriptome-wide scale. Next, we obtained the adjusted fold changes between IP and input samples in each stage, and these adjusted fold changes represent the relative m^6^A levels. We defined high confidence m^6^A-tagged genes with the thresholds: the TPM of gene in input samples > 5, the fold change in IP/input > 1.5, and adjusted *p* value < 0.05. To measure the correlation between m^6^A level and gene expression, we calculated the Pearson correlation coefficient between m^6^A level and gene expression, and the absolute value of the correlation coefficient > 0.5 and *p*-value < 0.05 was defined as m^6^A level significantly correlated with gene expression.

### Statistics and reproducibility

All experiments were carried out with at least three biological replicates and showed successful reproducibility. All graphs were generated using GraphPad Prism8 V.8.3.0.538 (64-bit). All data are shown as the mean with error bars representing the s.d. Two-tailed unpaired *t*-tests (Student’s *t*-test) were used to obtain the *p*-values. The following convention was used for indicating *p-*values: ns *p* > 0.05, * *p* < 0.05, ** *p* < 0.01, *** *p* < 0.001, **** *p* < 0.0001. Exact *p*-values are provided in Source data. The sample size (*n*) indicates the total number of independent biological replicates.

For immunoblotting and immunohistochemistry, representative images are shown. Each of these experiments was independently repeated at least three times.

### Reporting summary

Further information on research design is available in the Nature Research Reporting Summary linked to this article.

## Supplementary information

Supplementary Information

Reporting Summary

## Data Availability

Source data are provided with this paper in the Source Data file. The RNA-seq and m^6^A-RIP-seq data generated in this study have been deposited in the Gene Expression Omnibus (GEO) database under accession code GSE163964. Source data are provided with this paper.

## Source data

Source Data
