# Supplementary material for: N6-methyladenosine modification-mediated mRNA metabolism is essential for human pancreatic lineage specification and islet organogenesis
Source: Nat Commun. 2022 Jul 18;13:4148. doi: 10.1038/s41467-022-31698-2 (PMC9293889; doi:10.1038/s41467-022-31698-2)
Supplement: Supplementary file 1 — Supplementary Information [file 41467_2022_31698_MOESM1_ESM.pdf]

Title:

***N*^{6}-methyladenosine modification-mediated mRNA metabolism is essential for human pancreatic lineage specification and islet organogenesis**

Xiaojie Ma, et al.

Supplementary figures and legends

Supplementary Figure 1

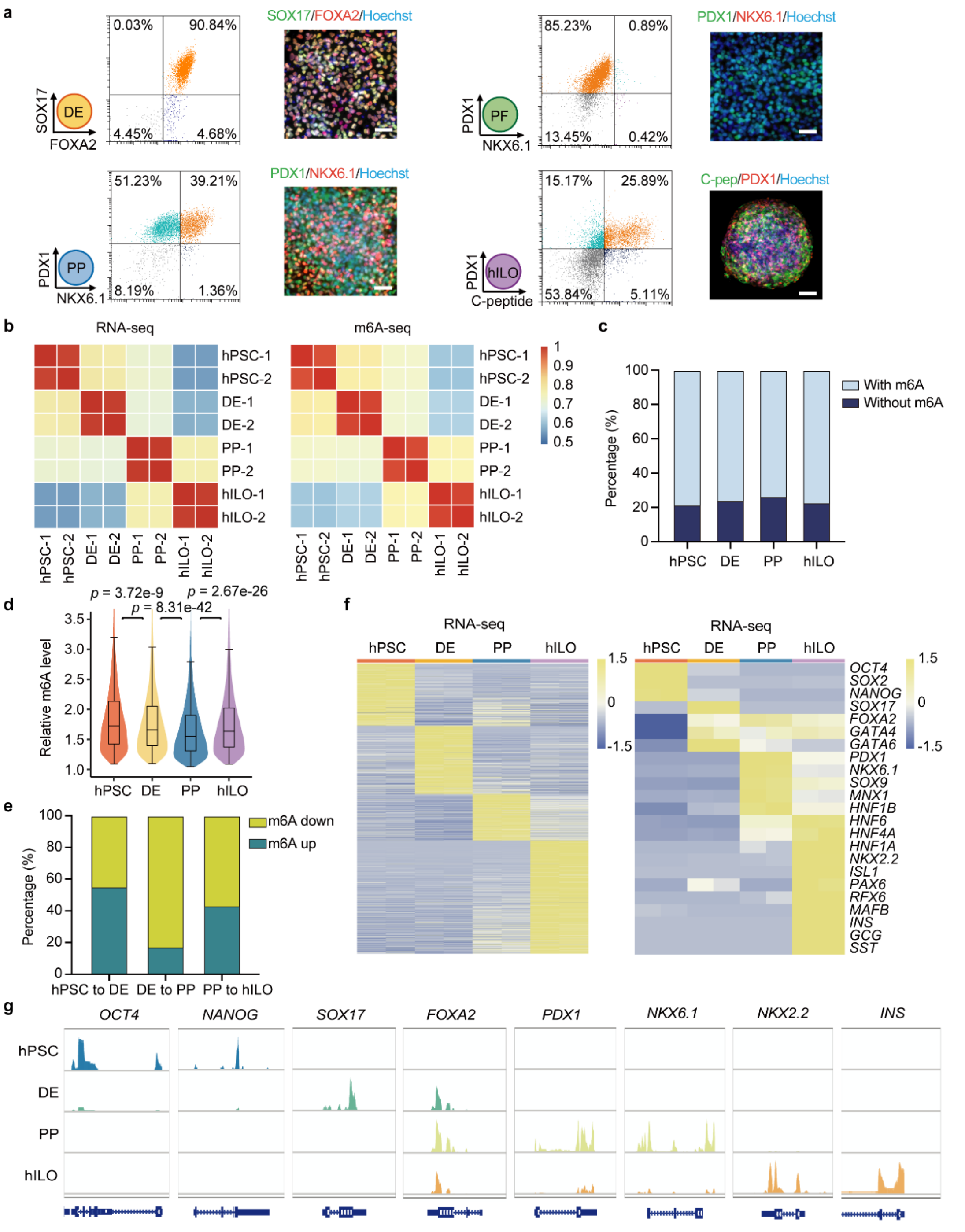

Supplementary Figure 1. Dynamic mRNA m^{6}A modification during pancreatic differentiation. a, Representative flow cytometry plots and immunostaining images of hPSC, DE, PP, and hILO cells with respective markers. Images are representative of three independent replicates. Scale bar, 100 µm. **b,** Spearman correlations of m^{6}A abundance and RNA expression profiles of hPSC, DE, PP, and hILO replicate samples.

c, Stacked histogram showing the percentage of genes with or without m^6A modification in hPSCs, DE, PPs, and hILOs. **d,** Violin-and-box plots displaying the relative m^6A levels in hPSCs, DE, PPs, and hILOs, $n = 2$. In box plots, the top and bottom lines represent the max and min values, lower and upper hinges represent first and third quartiles, the center line represents the median, and whiskers represent $\pm 1.5 \times$ the interquartile range, *p*-values were calculated using two-tailed Wilcoxon test. **e,** Stacked histogram showing the percentage of genes with decreasing or increasing m^6A levels from hPSCs to hILOs. **f,** Heatmap visualization of stage-specific gene expression in hPSCs, DE, PPs, and hILOs. **g,** Visualization of m^6A peaks on marker gene loci in hPSCs, DE, PPs, and hILOs. The sample size (*n*) indicates the total number of independent biological replicates.

Supplementary Figure 2

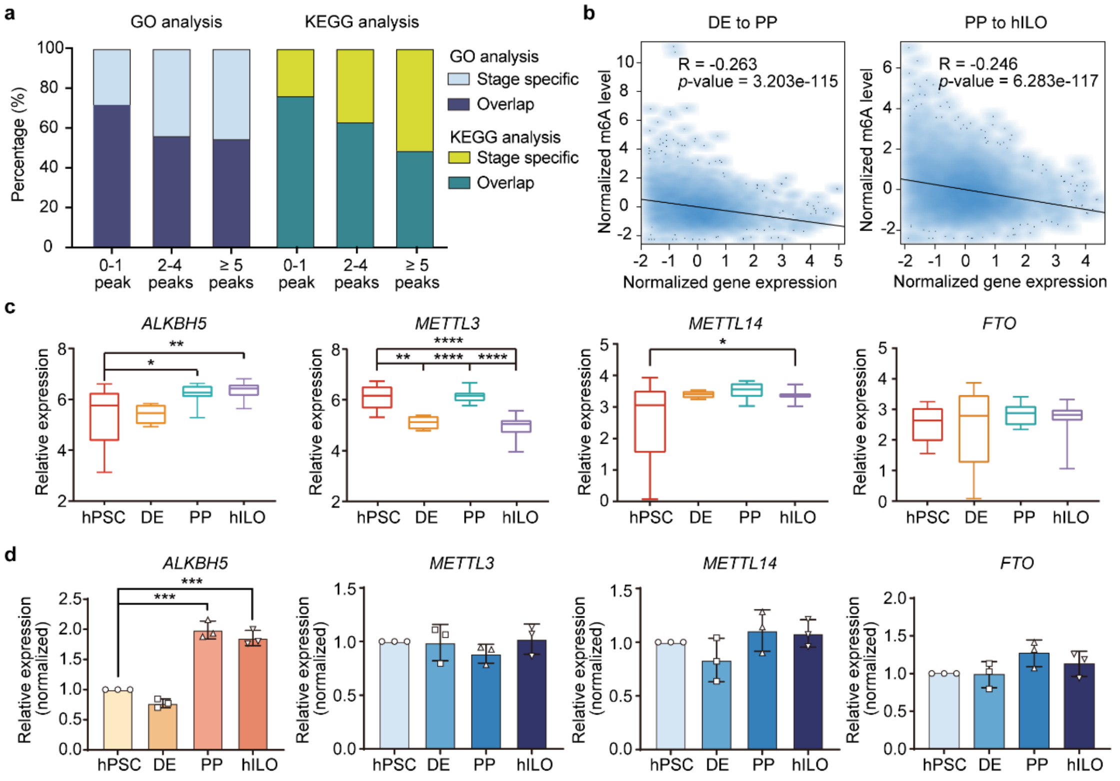

Supplementary Figure 2. Important role of mRNA m^{6}A modification across pancreatic differentiation. a, Stacked histogram showing the percentage of stage-specific and overlapped GO and KEGG terms enriched in genes with different numbers of m^{6}A peaks in hPSC, DE, PP, and hILO samples. **b,** Density scatter plots showing the correlation of gene expression and m6A level from DE to PPs and PPs to hILOs, p-values were calculated using two-tailed hypothesis test. **c,** Relative expression of *ALKBH5*, *METTL3*, *METTL14*, and *FTO* from previously published pancreatic differentiation datasets[1-7] (n = 41 biological samples). In box plots, the top and bottom lines represent the max and min values, lower and upper hinges represent first and third quartiles, the center line represents the median, and whiskers represent ±1.5 × the interquartile range. **d,** Gene expression of *ALKBH5*, *METTL3, METTL14*, and *FTO* during pancreatic differentiation (n = 3 biological replicates). All data are shown as mean ± s.d. Statistical significance calculated using two-tailed Student's t-test, * $p < 0.05$, ** $p < 0.01$, *** $p < 0.001$, **** $p < 0.0001$. Source data are provided as a Source Data file.

Supplementary Figure 3

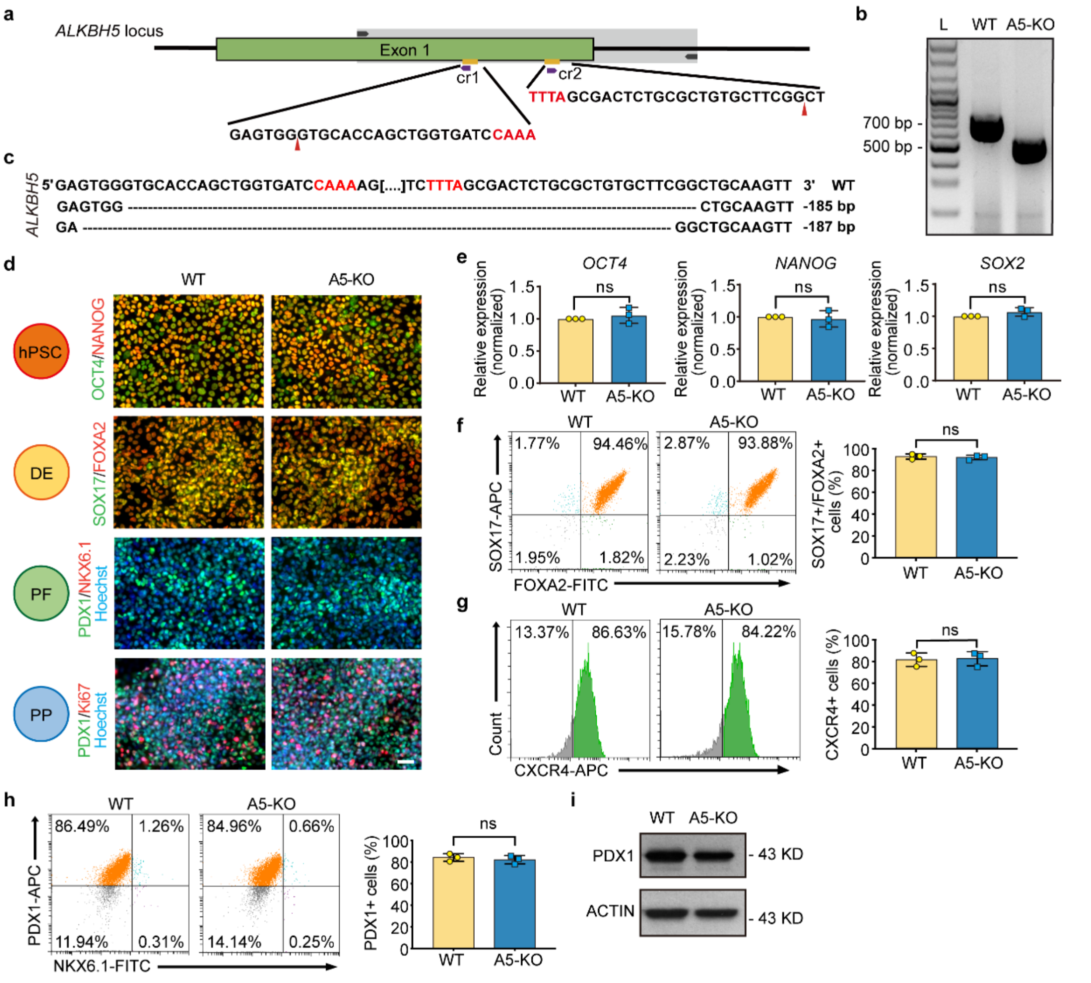

Supplementary Figure 3. ALKBH5 regulates pancreatic lineage specification. a, Schematic of Cpf1 crRNAs targeting sites at *ALKBH5* locus. Exon structures, green box; PCR amplicons, light gray box. crRNA targeting sequences are in bold; PAM sequences are in red. **b,** Genotyping of *ALKBH5* locus in WT and A5-KO hPSCs. Images are representative of three independent replicates. **c,** Sanger sequencing results of *ALKBH5* in WT, A5-KO, and A5-KO2 hPSCs. **d,** Immunofluorescent staining of important markers in WT and A5-KO cells on hPSC, DE, PF, and PP stages. Images are representative of three independent replicates. Scale bar, 100 μm. **e,** RT-qPCR analysis of *OCT4*, *SOX2*, and *NANOG* expression (n = 3 biological replicates). **f,** Representative flow cytometry and the percentage of SOX17^{+}FOXA2^{+} cells in WT and A5-KO DE populations (n = 3 biological replicates). **g,** Representative flow cytometry and the percentage of CXCR4^{+} cells in WT and A5-KO DE populations (n = 3 biological

replicates). **h,** Representative flow cytometry plots and the percentage of $PDX1^{+}NKX6.1^{-}$ cells in WT and A5-KO PF populations (*n* = 3 biological replicates). **i,** Representative western blotting of PDX1 in WT and A5-KO PPs. ACTIN was used as a loading control. Images are representative of three independent replicates. All data are expressed as mean ± s.d. Statistical significance calculated using two-tailed Student's *t*-test, ns $p > 0.05$. Source data are provided as a Source Data file.

Supplementary Figure 4

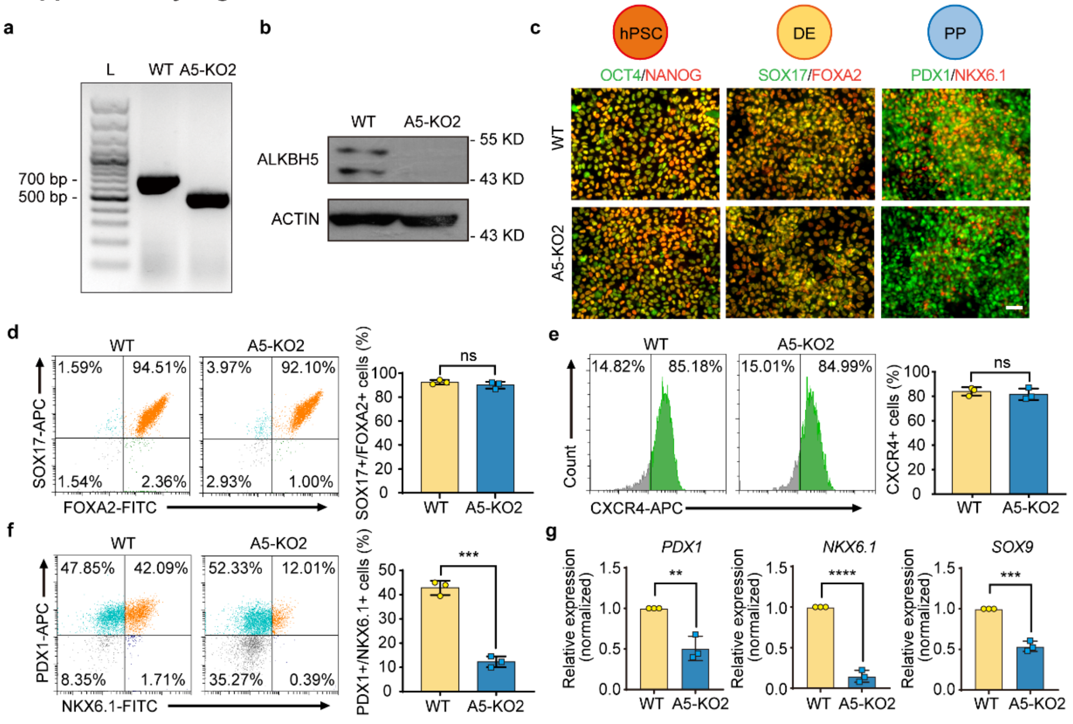

Supplementary Figure 4. Functional characterization of another A5-KO hPSC line. a, Genotyping of *ALKBH5* locus in WT and A5-KO2 hPSCs. Images are representative of three independent replicates. **b,** Western blotting result of ALKBH5 protein levels in WT and A5-KO2 hPSCs. Images are representative of three independent replicates. **c,** Immunofluorescent staining of important markers in WT and A5-KO2 cells on hPSC, DE, and PP stages. Images are representative of three independent replicates. Scale bar, 100 μm. **d,** Representative flow cytometry and the percentage of $SOX17^{+}FOXA2^{+}$ cells in WT and A5-KO2 DE populations (n = 3 biological replicates). **e,** Representative flow cytometry and the percentage of $CXCR4^{+}$ cells in WT and A5-KO2 DE populations (n = 3 biological replicates). **f,** Representative flow cytometry and the percentage of $PDX1^{+}NKX6.1^{+}$ cells in WT and A5-KO2 PP populations (n = 3 biological replicates). **g,** The expressions of PP marker genes *PDX1*, *NKX6.1*, and *SOX9* in WT and A5-KO2 PPs (n = 3 biological replicates). All data are shown as mean ± s.d. Statistical significance calculated using two-tailed Student's t-test, ns $p > 0.05$, ** $p < 0.01$, *** $p < 0.001$, **** $p < 0.0001$. Source data are provided as a Source Data file.

Supplementary Figure 5

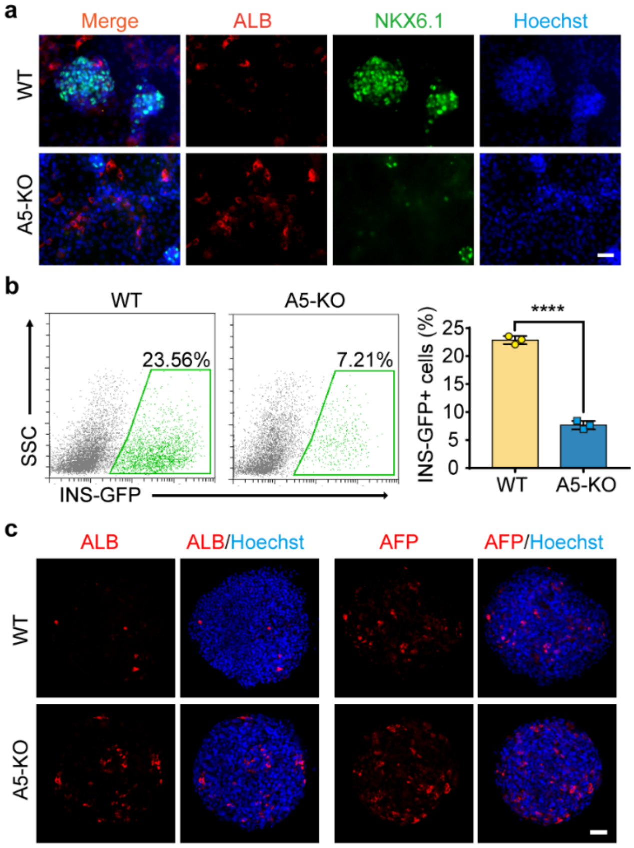

Supplementary Figure 5. ALKBH5 deletion disturbs pancreatic specification. a, Immunofluorescent staining of ALBUMIN, NKX6.1, and nuclei in WT and A5-KO PP cells. Images are representative of three independent replicates. Scale bar, 100 μm. **b,** Representative flow cytometry plots and the percentage of INS-GFP positive pancreatic β-like cells (n = 3 biological replicates). Data are expressed as mean ± s.d. Statistical significance calculated using two-tailed Student's t-test, **** $p < 0.0001$. **c,** Immunofluorescent staining of ALBUMIN (ALB), α-fetoprotein (AFP), and nuclei in WT and A5-KO hILOs. Images are representative of three independent replicates. Scale bar, 100 μm. Source data are provided as a Source Data file.

Supplementary Figure 6

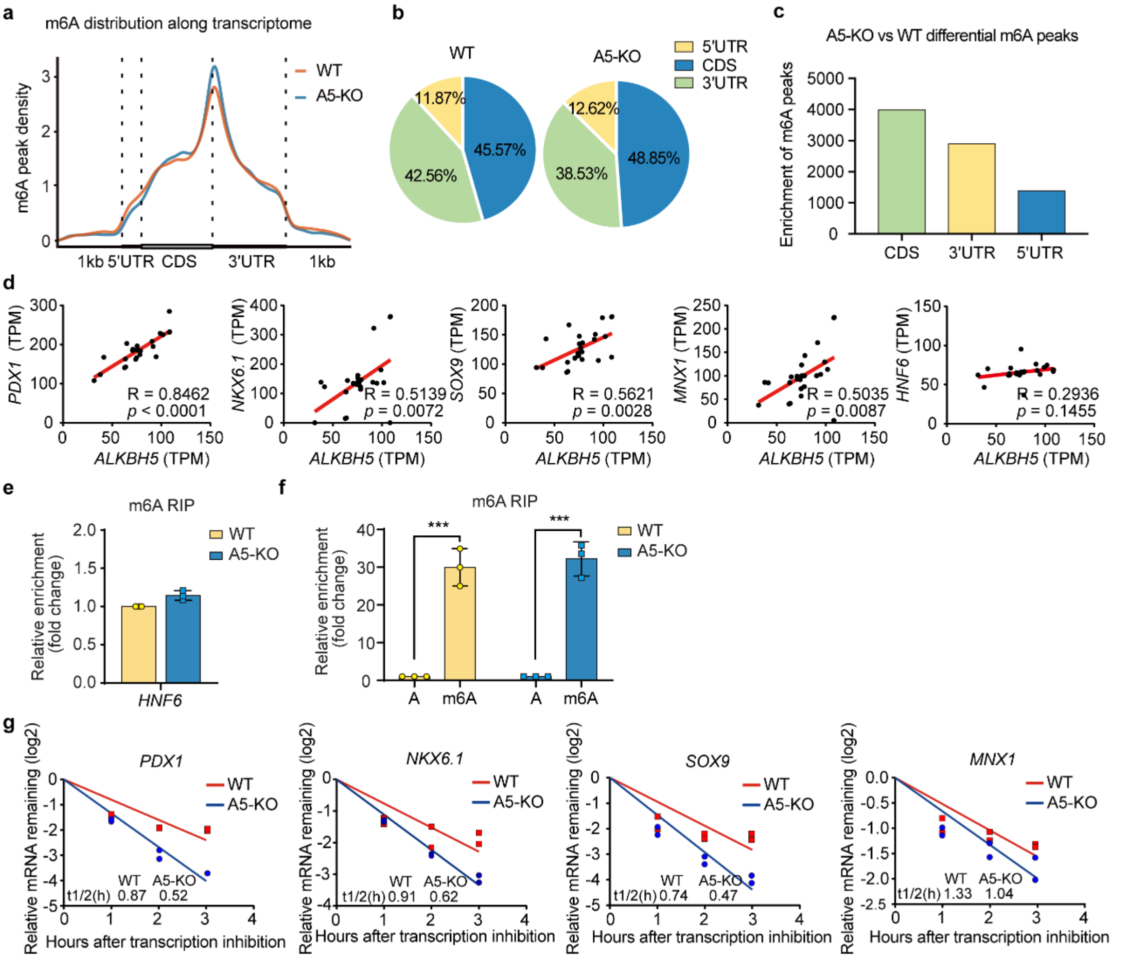

Supplementary Figure 6. ALKBH5 modulates the stability of target mRNA transcripts. a and **b,** m^{6}A peak distributions along transcripts in WT and A5-KO PPs. **c,** The distribution of differential m^{6}A peaks along transcripts. **d,** Scatter plots showing the Pearson correlations between the expression of *ALKBH5* and *PDX1*, *NKX6.1*, *SOX9*, *MNX1*, and *HNF6* from other published datasets[1-7] (n = 26 biological samples). The red lines represent fitting curves according to expression values of *ALKBH5* and pancreatic genes. **e,** RIP-qPCR of the m^{6}A enrichment on *HNF6* transcripts in WT and A5-KO PPs (n = 3 biological replicates). **f,** The negative and positive controls of m^{6}A-RIP-qPCR (n = 3 biological replicates). **g,** The decay curves for *PDX1*, *NKX6.1*, *SOX9*, and *MNX1* in WT and A5-KO PPs detected by RNA-seq (n = 2 biological replicates). All data are shown as mean ± s.d. Statistical significance calculated using two-tailed Student's *t*-test, *** $p < 0.001$. Source data are provided as a Source Data file.

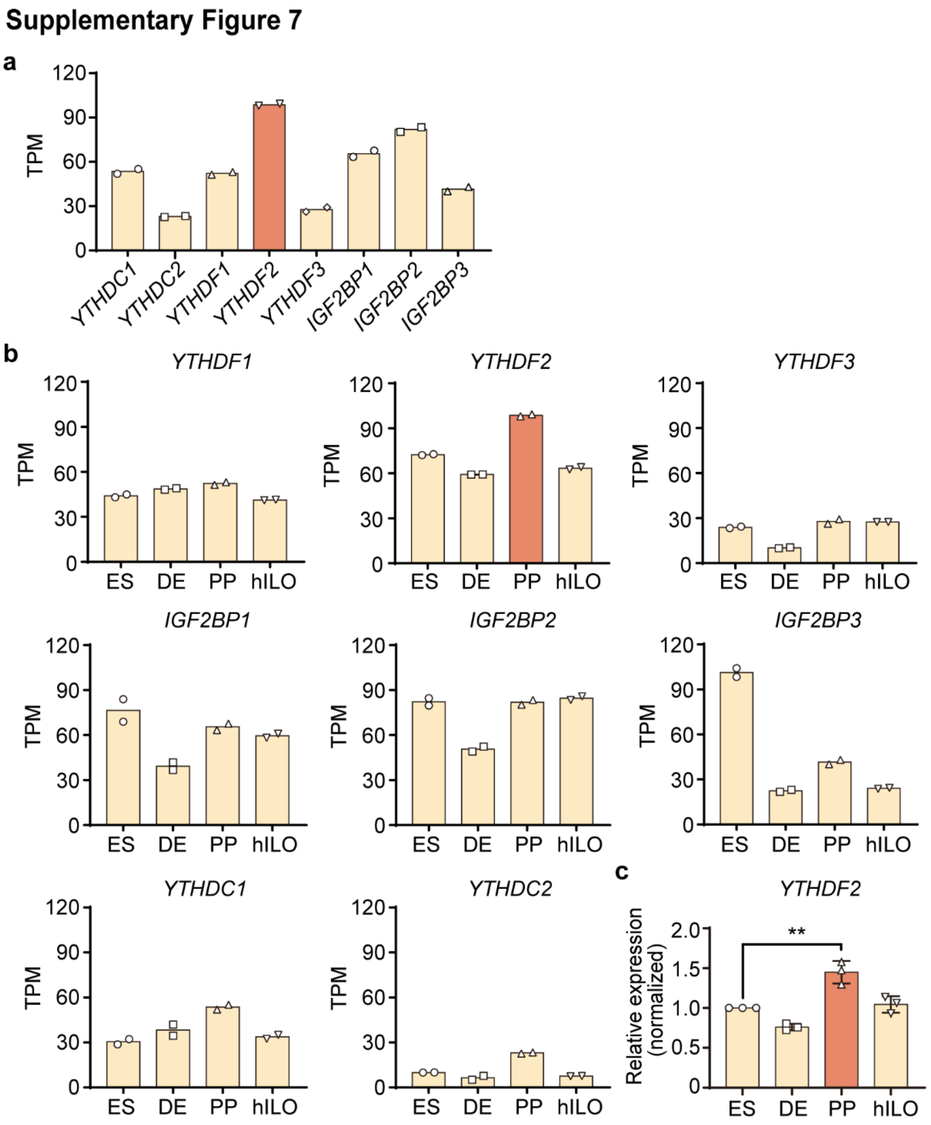

Supplementary Figure 7. Expression of m^{6}A readers during pancreatic differentiation. a, The expression of m^{6}A readers in PPs (n = 2 biological replicates). **b,** The expression of m^{6}A readers in hPSC, DE, PP, and hILO cells (n = 2 biological replicates). **c,** Gene expression of *YTHDF2* during pancreatic differentiation (n = 3 biological replicates). The data are shown as mean ± s.d. Statistical significance calculated using two-tailed Student's test, ** $p < 0.01$. Source data are provided as a Source Data file.

Supplementary Figure 8

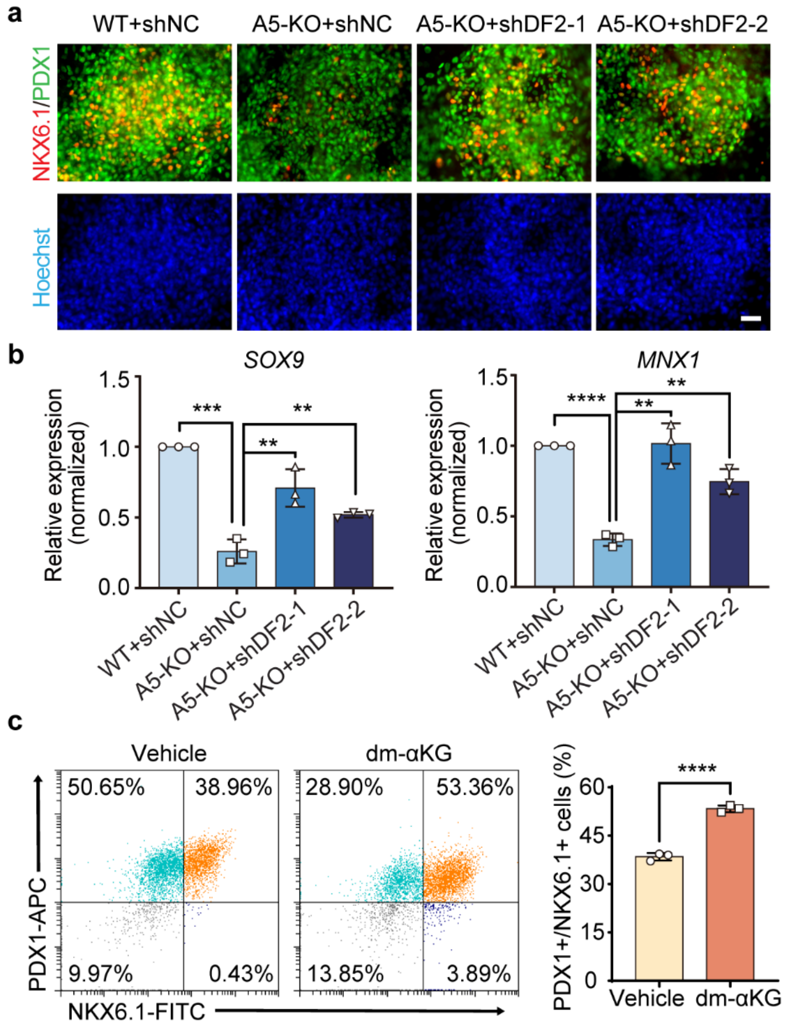

Supplementary Figure 8. YTHDF2 knock-down rescues and dm-αKG promotes human pancreatic differentiation. a, Immunofluorescent staining of WT+shNC, A5-KO+shNC and A5-KO+shDF2 PPs for PDX1, NKX6.1, and nuclei. Images are representative of three independent replicates. Scale bar, 100 μm. **b,** RT-qPCR analysis of *SOX9* and *MNX1* expression (n = 3 biological replicates). **c,** Representative flow cytometry plots and the percentage of PDX1^{+}NKX6.1^{+} cells in PP populations with or without dm-αKG (n = 3 biological replicates). All data are presented as mean ± s.d. Statistical significance calculated using two-tailed Student's t-test, ** $p < 0.01$, *** $p < 0.001$, **** $p < 0.0001$. Source data are provided as a Source Data file.

Supplementary Figure 9

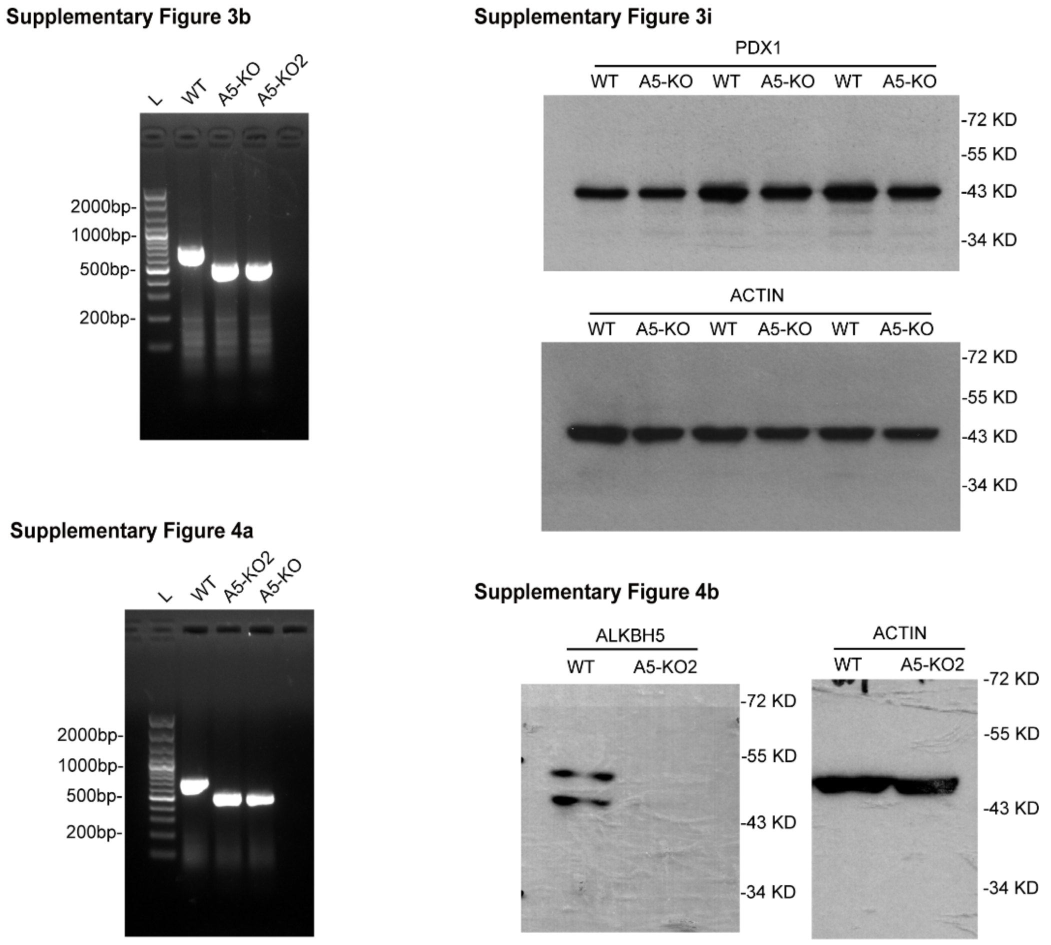

Supplementary Figure 9. Uncropped electrophoresis data and Western blots for Supplementary Figure 3b, 3i, 4a, and 4b.

SUPPLEMENTARY REFERENCES

1. Nair, G. G. et al. Recapitulating endocrine cell clustering in culture promotes maturation of human stem-cell-derived beta cells. *Nat. Cell Biol.* **21**, 263-274 (2019).
2. Hogrebe, N. J., Augsornworawat, P., Maxwell, K. G., Velazco-Cruz, L. & Millman, J. R. Targeting the cytoskeleton to direct pancreatic differentiation of human pluripotent stem cells. *Nat. Biotechnol.* **38**, 460-470 (2020).
3. Xie, R. et al. Dynamic chromatin remodeling mediated by polycomb proteins orchestrates pancreatic differentiation of human embryonic stem cells. *Cell Stem Cell* **12**, 224-237 (2013).
4. Alvarez-Dominguez, J. R. et al. Circadian entrainment triggers maturation of human In vitro Islets. *Cell Stem Cell* **26**, 108-122 (2020).
5. Amin, S. et al. Discovery of a drug candidate for GLIS3-associated diabetes. *Nat. Commun.* **9**, 2681 (2018).
6. Jiang, W., Liu, Y., Liu, R., Zhang, K. & Zhang, Y. The lncRNA DEANR1 facilitates human endoderm differentiation by activating FOXA2 expression. *Cell Rep.* **11**, 137-148 (2015).
7. Ghazizadeh, Z. et al. ROCKII inhibition promotes the maturation of human pancreatic beta-like cells. *Nat. Commun.* **8**, 298 (2017).

Title:

***N*⁶-methyladenosine modification-mediated mRNA metabolism is essential for human pancreatic lineage specification and islet organogenesis**

Xiaojie Ma, et al.

Supplementary tables

Supplementary Table 1. Summary information of key resources.

REAGENT or RESOURCE	SOURCE	IDENTIFIER
**Antibodies**		
Rabbit anti-ALKBH5 (1:1000)	Sigma	Cat#HPA007196
Mouse anti-β-ACTIN (1:10000)	Sungenebiotech	Cat#400-6210003
Goat anti-OCT4 (1:1000)	Santa cruz	Cat#sc-8629
Rabbit anti-NANOG (1:1000)	Abcam	Cat#Ab80892
Goat anti-SOX17 (1:1000)	R&D	Cat#AF1924
Rabbit anti-FOXA2 (1:1000)	Sigma-Aldrich	Cat#07-633
Goat anti-PDX1 (1:1000)	R&D SYSTEMS	Cat#AF2419
Mouse anti-NKX6.1 (1:500)	DSHB	Cat#F55A12
Rabbit anti-Ki67 (1:1000)	Abcam	Cat#ab15580
Goat anti-Somatostatin (SST) (1:1000)	Santa cruz	Cat#sc-7819
Goat anti-Glucagon (GCG) (1:1000)	Santa cruz	Cat#sc-7780
Rat anti-C-peptide (C-pep) (1:1000)	DSHB	Cat#GN-ID4
Goat anti-Albumin (ALB) (1:1000)	BETHYL	Cat#A80-129A
Rabbit anti-m^{6}A<br>Rabbit anti-m^{6}A	SYSY<br>Millipore	Cat#202003<br>Cat#ABE572
Rabbit anti-YTHDF2	Proteintech	Cat#24744-1-AP
APC-anti-CXCR4 (1:1000)	BioLegend	Cat#306509
HRP-goat anti-mouse IgG (1:5000)	EARTHOX	Cat#E030110-01
HRP-goat anti-rabbit IgG (1:5000)	EARTHOX	Cat#E030120-01
HRP-rabbit anti-goat IgG (1:5000)	EARTHOX	Cat#E030130-01
Alexa Fluor 488 donkey anti-mouse lgG (H+L) (1:2000)	Invitrogen	Cat#A-21202
Alexa Fluor 555 donkey anti-mouse lgG (H+L) (1:2000)	Invitrogen	Cat#A-31570
Alexa Fluor 555 donkey anti-rabbit lgG (H+L) (1:2000)	Invitrogen	Cat#A-31572
Alexa Fluor 555 donkey anti-goat lgG (H+L) (1:2000)	Invitrogen	Cat#A-21432

Alexa Fluor 488 donkey anti-rabbit lgG (H+L) (1:2000)	Invitrogen	Cat#A-21206
Alexa Fluor 488 donkey anti-goat lgG (H+L) (1:2000)	Invitrogen	Cat#A-11055
Alexa Fluor 647 donkey anti-goat lgG (H+L) (1:2000)	Invitrogen	Cat#A-21447
**Bacterial Strains**		
DH5a	ANGYUBIO	Cat#AYBIO-G6016
**Chemicals, Peptides, and Recombinant Proteins**		
Polybrene	Santa cruz	Cat#sc-134220
CHIR99021	TargetMol	Cat#T2310
Retinoic acid (RA)	Sigma	Cat#R2625
616452	TargetMol	Cat#T6337
3, 3', 5-Triiodo-l-thyronine sodium salt (T3)	Sigma	Cat#T6397
Vitamin C	Sigma-Aldrich	Cat#A8960-5G
N-acetyl-L-cysteine (NAC)	Sigma-Aldrich	Cat#A9165
LDN-193189	Tocris	Cat#6053
GDC-0449	Selleck	Cat#S1082
Compound E	MedChem Express	Cat#HY-14176
Thiazovivin	TargetMol	Cat#T2155
Trolox	Sigma	Cat#238813
Heparin	Sigma	Cat#H3149
Zinc sulfate	Sigma	Cat#Z0251
Human bFGF	Peprotech	Cat#100-18B
Human KGF	Peprotech	Cat#100-19
Human EGF	Peprotech	Cat#AF-100-15
Human/Murine/Rat Activin A	Peprotech	Cat#120-14P
Actinomycin D	Sigma	Cat#ab141058
DMEM basic	Gibco	Cat#C11995500CP
DMEM/F12	Gibco	Cat#11320033
RPMI 1640	Gibco	Cat#31870082
N2	Gibco	Cat#A1370701
B27	Gibco	Cat#0080085SA
BSA	Yeasen	Cat#36101ES25
Penicillin-Streptomycin	Gibco	Cat#15140-122
Non-essential amino acids	Gibco	Cat#11140-050
2-Mercaptoethanol	Sigma-Aldrich	Cat#M3148
Fetal bovine serum	Gibco	Cat#10270-106
Knockout serum replacement	Gibco	Cat#10828-028
Dimethyl-α-ketoglutarate (dm-αKG)	Sigma-Aldrich	Cat#349631
Shrimp alkaline phosphatase	NEB	Cat#M0371S
Trypsin-EDTA (0.05%)	Gibco	Cat#25300054
**Assay Kits**		
Quick-RNA MiniPrep Kit	ZYMO	Car#R1054

Endo-Free Plasmid Mini Kit	Omega	Cat#D6950
Agarose Gel DNA Extraction Kit	Easydo	Cat#0103050
PrimeScript RT Master Mix	Takara	Cat#RR036A
TB Green Premix Ex Taq II Kit	Takara	Cat#RR820A
Human Insulin Immunoassay Kit	EZassay	Cat#HM200
Gibson Assembly Kit	NEB	Cat#E2611L
Human Stem Cell Nucleofector Kit 1	Lonza	Cat#VPH-5012
SYBR Green Supermix	Bio-Rad	Cat# 1725120
TransDetect PCR Mycoplasma Detection Kit	TransGen Biotech	Cat#FM311-01
ZymoPURE Plasmid Maxiprep Kit	ZYMO	Cat#D4202
High Sensitivity ECL Chemiluminescence Detection Kit	Vazyme	Cat#E411-04
Super ECL Detection Reagent	Yeasen Biotech	Cat#36208ES60
Fast Pure Cell Total RNA Isolation Kit	Vazyme	Cat#RC101-01
GenElute mRNA Miniprep Kit	Sigma	Cat#MRN10–1KT
TRIzol™ Reagent	Invitrogen	Cat#15596018
NEBNext® rRNA Depletion Kit v2	NEB	Cat#E7400L
TruSeq® Stranded mRNA Library Prep	Illumina	Cat#20020594
SMARTer® Stranded Total RNA-Seq Kit v2	Takara	Cat#634411
**Deposited Data**		
RNA-seq data	This paper	GSE163945 [https://www.ncbi.nlm.nih.gov/geo/query/acc.cgi?acc=GSE163945]
m^{6}A-seq data	This paper	GSE163963 [https://www.ncbi.nlm.nih.gov/geo/query/acc.cgi?acc=GSE163963]
**Software and Algorithms**		
GraphPad Prism	GraphPad Software	http://www.graphpad.com
CHOPCHOP	N/A	http://chopchop.cbu.uib.no
SnapGene	N/A	https://www.snapgene.com/
Rstudio	N/A	https://rstudio.com/
Wave	Agilent	https://www.agilent.com/zh-cn/products/cell-analysis/software-download-for-wave-desktop
Fastp (v0.20.1)	N/A	https://github.com/OpenGene/fastp
HISAT2 (v2.1.0)	N/A	https://daehwankimlab.github.io/hisat2/

Stringtie (v2.0)	N/A	http://ccb.jhu.edu/software/stringtie/
Bowtie2 (v2.2.5)	N/A	http://bowtie-bio.sourceforge.net/bowtie2/index.shtml
HOMER (v4.11)	N/A	http://homer.ucsd.edu/homer/
SCIEX OS (v1.7.0)	N/A	https://sciex.com.cn/products/software/sciex-os-software

Supplementary Table 2. RT-qPCR primers.

Gene	Sequence
GAPDH-F	TGCACCACCAACTGCTTAGC
GAPDH-R	GGCATGGACTGTGGTCATGAG
FOXA2-F	GGGAGCGGTGAAGATGGA
FOXA2-R	TCATGTTGCTCACGGAGGAGTA
HNF6-F	ATGTCCAGCGTCGAACTCTAC
HNF6-R	TGCTTTGGTACAAGTGCTTGAT
ACTB-F	CACTCTTCCAGCCTTCCTTC
ACTB-R	GTACAGGTCTTTGCGGATGT
MNX1-F	GTTCAAGCTCAACAAGTACCTG
MNX1-R	CTTCTGTTTCTCCGCTTCCT
SOX9-F1	AGCTCTGGAGACTTCTGAACGAGAG
SOX9-R1	CGTTCTTCACCGACTTCCTCCGC
SOX9-F2	AGCGAACGCACATCAAGAC
SOX9-R2	CTGTAGGCGATCTGTTGGGG
PDX1-F1	CCTTTCCCATGGATGAAGTC
PDX1-R1	GAACTCCTTCTCCAGCTCTA
PDX1-F2	TGGAGCTGGCTGTCATGTTGA
PDX1-R2	CGCTTCTTGTCCTCCTCCTTTT
NKX6.1-F1	TCAACAGCTGCGTGATTTTC
NKX6.1-R1	CCAAGAAGAAGCAGGACTCG
NKX6.1-F2	GGGAGGGAGGGGCTACAATA
NKX6.1-R2	ATGTTTCAGACCCAGAGCGG
SOX17-F	ATTTCCTCGGTGGTGTCC
SOX17-R	CCAAACTGTTCAAGTGGCAGA
OCT4-F	GAGAAGGAGAAGCTGGAGCA
OCT4-R	AATAGAACCCCCAGGGTGAG
NANOG-F	GATTTGTGGGCCTGAAGAAA
NANOG-R	CAGATCCATGGAGGAAGGAA
SOX2-F	CATGGACAGTTACGCGCACAT
SOX2-R	AGTTGTACTGCAGGGCGCTCA
YTHDF2-F	AGGACAAATGGAAGGGTCGT
YTHDF2-R	GGAAGTGGTGTGCTTGTAGC
ALKBH5-F	TCGGCGAAGGCTACACTTAC
ALKBH5-R	TGGTAGTCGTTGATGACGGC
METTL3-F	TTGTCTCCAACCTTCCGTAGT
METTL3-R	CCAGATCAGAGAGGTGGTGTAG
METTL14-F	AAATGCTGGACTTGGGATGATA
METTL14-R	CCCATTTTCGTAAACACACTCTT
FTO-F	TGAGGATGCTGTGCCATTGT

FTO-R	ACGTTGTATGCTGCTCTGCT
NKX6.1-RIP-F	GAAGTTGTGGGAGGGGATGT
NKX6.1-RIP-R	CGCAGTGCATTTGGTGGTC
PDX1-RIP-F	GTTCCGAGGTAGAGGCTGTG
PDX1-RIP-R	TGAAGGTCATACTGGCTCGTG
SOX9-RIP-F	AAAGCGGAGCTCGAAACTGAC
SOX9-RIP-R	AAGTTTCCGGGGTTGAAACTG
MNX1-RIP-F	CACTCGCGTGGGAGTTTGTG
MNX1-RIP-R	TGCCAATAATCAAAGTCGCCG
GAPDH-RIP-F1	CCACATCGCTCAGACACCAT
GAPDH-RIP-R1	TGAAGGGGTCATTGATGGCAA
HNF6-RIP-F	GGTGTGTAGCACAGACGAGT
HNF6-RIP-R	TTTGCAGCTCTCACTGGGAC

Supplementary Table 3. PCR primers for genotyping.

**Gene**	**Sequence**
ALKBH5-KO-F	CTCTTCAGCCAGGACGAGTG
ALKBH5-KO-R	CGGGGACAGGATTTCCACAT
