# Supplementary material for: N6-methyladenosine modification-mediated mRNA metabolism is essential for human pancreatic lineage specification and islet organogenesis
Source: Nat Commun. 2022 Jul 18;13:4148. doi: 10.1038/s41467-022-31698-2 (PMC9293889; doi:10.1038/s41467-022-31698-2)
Supplement: Supplementary file 2 — Reporting Summary [file 41467_2022_31698_MOESM2_ESM.pdf]

nature portfolio

Corresponding author(s): Saiyong Zhu, Jianzhao Liu

Last updated by author(s): Apr 20, 2022

Reporting Summary

Nature Portfolio wishes to improve the reproducibility of the work that we publish. This form provides structure for consistency and transparency in reporting. For further information on Nature Portfolio policies, see our Editorial Policies and the Editorial Policy Checklist.

Statistics

For all statistical analyses, confirm that the following items are present in the figure legend, table legend, main text, or Methods section.

n/a	Confirmed	
☐	☒	The exact sample size (*n*) for each experimental group/condition, given as a discrete number and unit of measurement
☐	☒	A statement on whether measurements were taken from distinct samples or whether the same sample was measured repeatedly
☐	☒	The statistical test(s) used AND whether they are one- or two-sided<br>*Only common tests should be described solely by name; describe more complex techniques in the Methods section.*
☒	☐	A description of all covariates tested
☐	☒	A description of any assumptions or corrections, such as tests of normality and adjustment for multiple comparisons
☐	☒	A full description of the statistical parameters including central tendency (e.g. means) or other basic estimates (e.g. regression coefficient) AND variation (e.g. standard deviation) or associated estimates of uncertainty (e.g. confidence intervals)
☐	☒	For null hypothesis testing, the test statistic (e.g. *F*, *t*, *r*) with confidence intervals, effect sizes, degrees of freedom and *P* value noted<br>*Give P values as exact values whenever suitable.*
☒	☐	For Bayesian analysis, information on the choice of priors and Markov chain Monte Carlo settings
☐	☒	For hierarchical and complex designs, identification of the appropriate level for tests and full reporting of outcomes
☐	☒	Estimates of effect sizes (e.g. Cohen's *d*, Pearson's *r*), indicating how they were calculated

Our web collection on statistics for biologists contains articles on many of the points above.

Software and code

Policy information about availability of computer code

Data collection	Images of agarose gel was collected by JS-2000 gel imaging analyzer (Peiqing, China).<br>The qPCR data were acquired by CFX96 Connect Real-Time PCR machine (Bio-Rad, USA).<br>Western blots image was collected using the OPTIMAX X-Ray Film Processor (PROTEC, Germany).<br>The fluorescence image was taken using Axio Observer 3 and LSM880 confocal microscopes (Zeiss, Germany).<br>The bright field iamge was taken through a microscope (Olympus, CKX41, Japan).<br>Mass spectrometry data was collected by UHPLC-QQQ-MS/MS (AB SCIEX, USA).<br>The FACS data was acquired by Beckman CytoFlex S (Beckman, USA).<br>High through-put sequencing data in this study was collected by Illumina HiSeq 2500 system.<br>Other pancreatic differentiation datasets were downloaded from NCBI GEO database (https://www.ncbi.nlm.nih.gov/geo/) and EBI ArrayExpress database(https://www.ebi.ac.uk/arrayexpress/).
Data analysis	The FACS data was acquired by Beckman CytoFlex S and analyzed by CytExpert software.<br>Data analysis and statistics were performed in GraphPad Prism, version 8.<br>Fastp (v0.20.1) was used to trim adapter and low quality reads of raw reads.<br>HISAT2 (v2.1.0) was used to map reads to the human genome (GRCh38).<br>Stringtie (v2.0) was used to perform transcript assembly.<br>HOMER (v4.11) was used for motif enrichment analysis.<br>The R package DESeq2 was used to perform differential expression analysis.<br>The R package pheatmap was used to generate heatmaps.<br>The R package exomePeak2 was used to identify m6A peaks.

For manuscripts utilizing custom algorithms or software that are central to the research but not yet described in published literature, software must be made available to editors and reviewers. We strongly encourage code deposition in a community repository (e.g. GitHub). See the Nature Portfolio guidelines for submitting code & software for further information.

Data

Policy information about availability of data

All manuscripts must include a data availability statement. This statement should provide the following information, where applicable:

- Accession codes, unique identifiers, or web links for publicly available datasets
- A description of any restrictions on data availability
- For clinical datasets or third party data, please ensure that the statement adheres to our policy

All sequencing data generated in this study are available from the Gene Expression Omnibus under the accession ID GSE163964. All other data generated is included as Supplementary or Source Data. A list of publicy avaliable datasets used in the study can ba found from NCBI GEO database (GSE10979; GSE137659; GSE139817; GSE114051; GSE44875 and GSE84325) and EBI ArrayExpress database(E-MTAB-1086).

Field-specific reporting

Please select the one below that is the best fit for your research. If you are not sure, read the appropriate sections before making your selection.

☒ Life sciences ☐ Behavioural & social sciences ☐ Ecological, evolutionary & environmental sciences

For a reference copy of the document with all sections, see nature.com/documents/nr-reporting-summary-flat.pdf

Life sciences study design

All studies must disclose on these points even when the disclosure is negative.

Sample size	Sample size was chosen based upon previous studies in the field, the technical difficulty and throughput of the assay. The sample size (n) of each experiment is indicated in the figure legends in the main manuscript and supplementary information files. At least 3 biological replicates was used in this study which show the reproducibility of the data and the detection of significant changes can support meaningful conclusion.
Data exclusions	No data points were excluded fron analysis in any experiment depicted in this manuscript.
Replication	The findings in this paper were remarkably reproducible. Every experiment was performed multiple times and the reproducibility was stated in the main manuscript.
Randomization	For cell culture based experiments all wells in each biological replicate were split from the same batch of the cells and randomly divided for each treatment.
Blinding	The investigators were not blinded to allocation during experiments and outcome assessment.

Reporting for specific materials, systems and methods

We require information from authors about some types of materials, experimental systems and methods used in many studies. Here, indicate whether each material, system or method listed is relevant to your study. If you are not sure if a list item applies to your research, read the appropriate section before selecting a response.

Materials & experimental systems

n/a	Involved in the study	
☐	☒	Antibodies
☐	☒	Eukaryotic cell lines
☒	☐	Palaeontology and archaeology
☒	☐	Animals and other organisms
☒	☐	Human research participants
☒	☐	Clinical data
☒	☐	Dual use research of concern

Methods

n/a	Involved in the study	
☒	☐	ChIP-seq
☐	☒	Flow cytometry
☒	☐	MRI-based neuroimaging

Antibodies

Antibodies used

All antibodies used in the study are listed in KEY RESOURCES TABLE.
Here we state in order: Antibody name (dilution, Company; Catalogue number)
Rabbit anti-ALKBH5 (1:1000, Sigma, Cat#HPA007196)
Mouse anti-β-ACTIN (1:10000, Sungenebiotech, Cat#400-6210003)
Goat anti-OCT4 (1:1000, Santa cruz, Cat#sc-8629)
Rabbit anti-NANOG (1:1000, Abcam, Cat#Ab80892)

Goat anti-SOX17 (1:1000, R&D, Cat#AF1924)
Rabbit anti-FOXA2 (1:1000, Sigma-Aldrich, Cat#07-633)
Goat anti-PDX1 (1:1000, R&D SYSTEMS, Cat#AF2419)
Mouse anti-NKX6.1 (1:500, DSHB, Cat#F55A12)
Rabbit anti-Ki67 (1:1000, Abcam, Cat#ab15580)
Goat anti-Somatostatin (SST) (1:1000, Santa cruz, Cat#sc-7819)
Goat anti-Glucagon (GCG) (1:1000, Santa cruz, Cat#sc-7780)
Rat anti-C-peptide (C-pep) (1:1000, DSHB, Cat#GN-ID4)
Goat anti-Albumin (ALB) (1:1000, BETHYL, Cat#A80-129A)
Rabbit anti-m6A (5ug, SYSY, Cat#202003)
Rabbit anti-m6A (5ug, Millipore, Cat#ABE572)
Rabbit anti-YTHDF2 (5ug, Proteintech, Cat#24744-1-AP)
APC-anti-CXCR4 (1:1000, BioLengend, Cat#306509)
HRP-goat anti-mouse IgG (1:5000, EARTHOX, Cat#E030110-01)
HRP-goat anti-rabbit IgG (1:5000, EARTHOX, Cat#E030120-01)
HRP-rabbit anti-goat IgG (1:5000, EARTHOX, Cat#E030130-01)
Alexa Fluor 488 donkey anti-mouse IgG (H+L) (1:2000, Invitrogen, Cat#A-21202)
Alexa Fluor 555 donkey anti-mouse IgG (H+L) (1:2000, Invitrogen, Cat#A-31570)
Alexa Fluor 555 donkey anti-rabbit IgG (H+L) (1:2000, Invitrogen, Cat#A-31572)
Alexa Fluor 555 donkey anti-goat IgG (H+L) (1:2000, Invitrogen, Cat#A-21432)
Alexa Fluor 488 donkey anti-rabbit IgG (H+L) (1:2000, Invitrogen, Cat#A-21206)
Alexa Fluor 488 donkey anti-goat IgG (H+L) (1:2000, Invitrogen, Cat#A-11055)
Alexa Fluor 647 donkey anti-goat IgG (H+L) (1:2000, Invitrogen, Cat#A-21447)

Validation

All of the antibodies used in this study were commercial and suitable for specific purposes. The antibodies were validated based on the information from the manufacturer's instructions and was supported by multiple publications. For key essential antibody ALKBH5, we further validated by western blotting upon depletion of the proteins. In addition, for the different stage cell markers like OCT4, SOX17, PDX1 and C-pep, we further validated by immunostaining and FACS. And anti-m6A and YTHDF2 antibody were used for RIP experiment.

Eukaryotic cell lines

Policy information about cell lines

Cell line source(s)	MEL1 INSGFP/W hESC line was a kind gift from Drs. E. G. Stanley and Andrew Elefanty. HEK293T cells were purchased from ATCC (CRL-3216).
Authentication	HEK293T cells were authenticated with morphology, karyotyping, and PCR based approaches by ATCC.
Mycoplasma contamination	Mycoplasma contamination was negative by routine check.
Commonly misidentified lines (See ICLAC register)	No commonly misidentified cell lines were used in this study.

Flow Cytometry

Plots

Confirm that:

- [x] The axis labels state the marker and fluorochrome used (e.g. CD4-FITC).
- [x] The axis scales are clearly visible. Include numbers along axes only for bottom left plot of group (a 'group' is an analysis of identical markers).
- [x] All plots are contour plots with outliers or pseudocolor plots.
- [x] A numerical value for number of cells or percentage (with statistics) is provided.

Methodology

Sample preparation	Cells were dissociated into single cells using Accutase and washed with PBS buffer. Then, cells were fixed by 4% PFA for 30 min and washed by PBST for 3 to 5 times followed by centrifuged at 1500 rpm, 5 min per time. Thereafter, cells were blocked with blocking buffer and incubated with primary antibodies at 4°C overnight. After washing with PBST for 3 times, cells were incubated in secondary antibodies at RT for 1 hour. All the antibodies are detailed in KEY RESOURCES TABLE. FACS data were acquired by Beckman CytoFlex (Beckman) and analyzed by CytExpert software.
Instrument	Beckman CytoFlex
Software	CytExpert
Cell population abundance	No sorting in this study.

Gating strategy	Gating was performed using the negative cell populations without primary antibody incubation. Gating to quantify positive and negative populations were applied uniformly to all samples.

☒ Tick this box to confirm that a figure exemplifying the gating strategy is provided in the Supplementary Information.
